# Plant Vascular Cell Division Is Maintained by an Interaction between PXY and Ethylene Signalling

**DOI:** 10.1371/journal.pgen.1002997

**Published:** 2012-11-15

**Authors:** J. Peter Etchells, Claire M. Provost, Simon R. Turner

**Affiliations:** Faculty of Life Sciences, University of Manchester, Manchester, United Kingdom; Carnegie Institution of Washington, United States of America

## Abstract

The procambium and cambium are meristematic tissues from which vascular tissue is derived. Vascular initials differentiate into phloem towards the outside of the stem and xylem towards the inside. A small peptide derived from *CLV-3/ESR1-LIKE 41* (*CLE41*) is thought to promote cell divisions in vascular meristems by signalling through the PHLOEM INTERCALLATED WITH XYLEM (PXY) receptor kinase. *pxy* mutants, however, display only small reductions in vascular cell number, suggesting a mechanism exists that allows plants to compensate for the absence of PXY. Consistent with this idea, we identify a large number of genes specifically upregulated in *pxy* mutants, including several AP2/ERF transcription factors. These transcription factors are required for normal cell division in the cambium and procambium. These same transcription factors are also upregulated by ethylene and in ethylene-overproducing *eto1* mutants. *eto1* mutants also exhibit an increase in vascular cell division that is dependent upon the function of at least 2 of these *ERF* genes. Furthermore, blocking ethylene signalling using a variety of ethylene insensitive mutants such as *ein2* enhances the cell division defect of *pxy*. Our results suggest that these factors define a novel pathway that acts in parallel to PXY/CLE41 to regulate cell division in developing vascular tissue. We propose a model whereby vascular cell division is regulated both by PXY signalling and ethylene/ERF signalling. Under normal circumstances, however, PXY signalling acts to repress the ethylene/ERF pathway.

## Author Summary

Plants transport water and nutrients throughout their bodies using a specialised vascular system. Vascular tissue is also responsible for providing structural support to plants; for example, wood is made up of specialised vascular cells. Consequently, the vascular system constitutes the majority of plant biomass. Chemicals from plant biomass could be used to make the next generation of biofuels in order to reduce dependence on fossil fuels. Vascular tissue is derived from a group of dividing cells present in a structure called the procambium, but mechanisms controlling cell division in this structure remain poorly understood. Understanding the events that occur in the procambium may help us to understand how we can best utilise plants for increased plant biomass, for example, for biofuel and wood production. We have identified a number of genes that regulate cell division in the procambium that are controlled by the gaseous plant hormone ethylene. We show that ethylene signalling, in turn, interacts with *PXY*, a gene encoding a signalling component that also controls vascular cell division. Our results demonstrate that the interaction between ethylene and *PXY* signalling is responsible for maintaining the plant vascular system.

## Introduction

Organised cell division and differentiation are required throughout nature for development of ordered body plans. The annual rings of trees which result from seasonal differences in radial growth are a widely recognisable example of the highly regulated nature of this process. Radial growth is achieved by generation of new vascular tissue that occurs via ordered cell divisions in the vascular meristem known as the cambium. Divisions in the cambium result in displacement of older cells to its periphery where they subsequently differentiate into xylem towards the inside of the stem or phloem towards the outside. Cambial cells divide in a highly ordered manner along their long axis giving rise to files of cells in a process that is most apparent in the growth rings of trees but also apparent in most higher plants such as *Arabidopsis* [1]. The ordered nature of this cell division is required for vascular tissue organisation and consequently is essential for both primary and secondary vascular development [2].

The receptor kinase *PHLOEM INTERCALATED WITH XYLEM* (*PXY*) was identified as being essential for ordered, coordinated cell divisions in the procambium [2] and has been shown to bind a peptide derived from CLV-3/ESR1-LIKE 41 (CLE41) and CLE44 [3], which was originally identified as TDIF, a peptide that represses tracheary element formation in transdifferentiation assays [4]. *CLE41*, and related *CLE42* [5], [6] also function through the PXY receptor to provide positional information required for orientation of the cell division plane in the procambium [7]. *CLE41* and *CLE42* over-expression lines have more cells in vascular bundles than those of wild type counterparts [7] and an increased diameter of the hypocotyl vascular cylinder [3], [8]. These increases in vascular cell number and hypocotyl diameter are completely abolished in *pxy 35S::CLE41* and *pxy 35S::CLE42* lines [7]. Consequently, *CLE41/42* induced vascular cell divisions occur in a *PXY* dependent manner demonstrating that PXY signalling, in addition to setting the division plane, also promotes the divisions themselves [3], [7]. A downstream target of PXY, the *WUSCHEL*-*RELATED HOMEOBOX* (*WOX*) gene, *WOX4* is thought to be required for the promotion of these divisions [9] and *wox4* mutants have been shown to have defects in vascular proliferation [10], [11].

Given that PXY signalling promotes vascular cell division, it might be expected that *pxy* mutants demonstrate a reduction in cell division, however in inflorescence stems of 5 week old plants no defects in the rate of cell division were reported [2]. Furthermore, *pxy* mutant hypocotyls exhibit only a small reduction in diameter at senescence suggesting only a small decrease in the total number of vascular cell divisions [7]. One explanation for this apparent contradiction is that a compensatory pathway exists that may be activated in the absence of *pxy*.

The gaseous hormone ethylene, has been shown to promote radial growth in several tree species [12], [13], [14], and more recently, radial growth and increased cambial cell division in tension wood of poplar was shown to be ethylene-induced [15]. Here we demonstrate that *pxy* and *wox4* work together with several *ETHYLENE RESONSE FACTOR* (*ERF*) transcription factors and ethylene signalling to regulate cell divisions during *Arabidopsis* vascular development. We propose that in *pxy* mutants, cell numbers are maintained by the up-regulation of an ethylene pathway that increases expression of these *ERFs*. We present evidence for a model whereby vascular cell division is promoted by an interaction between PXY and ethylene signalling. Consequently, in addition to its role in mediating stress responses including the development of tension wood [15], our results suggest a more general role for ethylene in regulation of vascular cell division.

## Results

### Ethylene Response Factors are upregulated in *pxy* mutants

There are apparent contradictory observations with regard to the role of PXY/CLE41 in the regulation of the rate of vascular cell division. While *CLE41* overexpression results in more cells [7], loss of PXY has little effect on vascular cell number [2]. One possible explanation is that an alternative pathway that also promotes vascular cell division is upregulated in *pxy* mutant plants. To test this hypothesis, we generated microarray expression data for the central part of *pxy-3* mutant inflorescence stems and compared it to comparable data from wild type (Experiment E-MEXP-2420, http://www.ebi.ac.uk/arrayexpress). Intriguingly 12 members of the AP2/ERF family of transcription factors, predominantly from classes VIII-X [16] were found to be expressed at higher levels in *pxy* than wild type (Table S1). *ERF109* (At4g34410; also known as *RRTF* [17]), *ERF11* (At1g28370), *ERF104* (At5g61600), and *ERF018* (At1g74930; also known as *ORA47* [18]) were increased 4.3, 3.0, 2.8 and 2.8, -fold, respectively (Table S1). Four further *AP2/ERF* family members *AtERF1* (At4g17500), *ERF2* (At5g47220), *ERF5* (At5g47230), and *ERF6* (At4g17490) demonstrated between 1.5 and 2-fold increases in expression. To confirm that the expression changes identified in array experiments were robust, we used qRT-PCR to retest expression levels of *ERF018*, *ERF109* and *AtERF1* in wild type and *pxy-3* plants using RNA isolated from similar tissue to that used in microarrays. We observed similar fold changes in qRT-PCR to those previously identified in arrays when relative expression levels were normalised to that of *ACT2* or *18s rRNA* (Figure S1).

**Figure 1:**
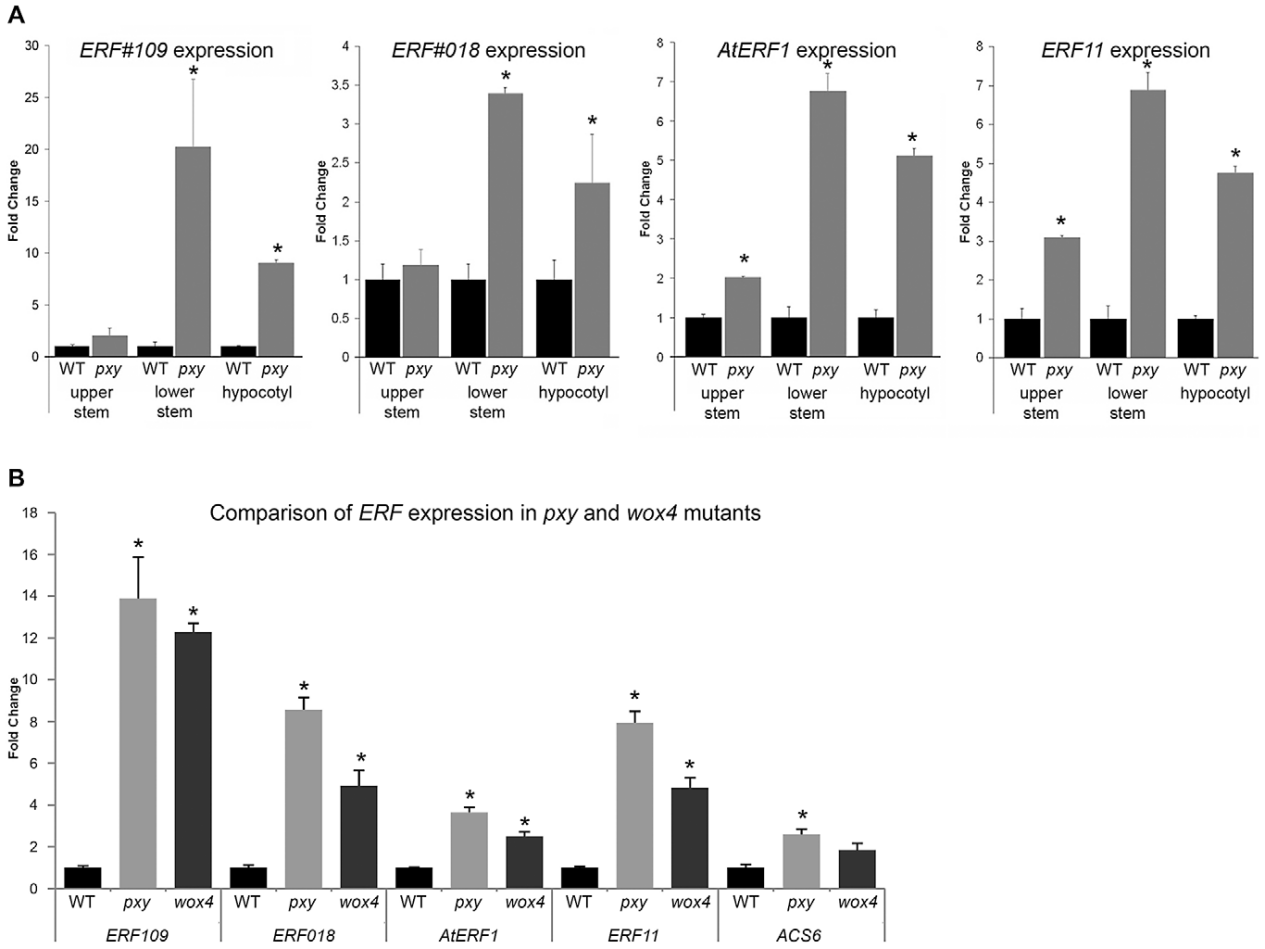
Expression of *ERF* transcription factors in Col, *pxy*, and *wox4* mutants. (A) qRT-PCR's showing *ERF* expression changes in upper inflorescence stem, lower inflorescence stem and hypocotyl of 5 week old plants, normalised to *18SrRNA*. In all instances, higher *ERF* expression was observed in *pxy* mutants (grey) than wild type (black) for hypocotyls and stem bases. *AtERF1* and *ERF11* demonstrated expression increases in the upper inflorescence stem. (B) qRT-PCR's showing *ERF* expression changes in lower half of inflorescence stems of 5 week old plants, normalised to *18SrRNA*. In all cases, expression was higher (p<0.0001) in *pxy* mutants (light grey bars) and *wox4* (dark grey bars) than wild type (black). Error bars show standard error. *represents expression significantly different from wild type controls (*p*<0.0001). Samples were measured in technical triplicates on biological triplicates.

*Arabidopsis* inflorescence stems represent a developmental series as vascular tissue at the top of stems is newly initiated in contrast to more mature vasculature at the base of stems. To further investigate the expression pattern of genes differentially expressed in *pxy*, we assayed expression of four of the most upregulated *ERF*'s (*AtERF1*, *ERF11*, *ERF109* and *ERF018*) at both the top (2–4 cm below the shoot apex) and the base (1–3 cm above the rosette) of inflorescence stems from 5 week old plants using qRT-PCR (Figure 1A). The base of stems demonstrated larger fold changes in gene expression in *pxy* than was observed in the middle of stems (Table S1; Figure S1) as *ERF109*, *ERF11*, *AtERF1* and *ERF018* expression was increased 20, 7, 7 and 3-fold, respectively. In contrast, at the top of stems significant changes were only observed for *AtERF1* and *ERF11* suggesting that expression of these genes is upregulated in newly formed *pxy* mutant stems and this upregulation is progressively increased as vascular tissue matures (Figure 1A). Similar increases in *ERF* expression were also observed in *pxy* hypocotyls compared to wild type counterparts (Figure 1A).

*WOX4* has been placed in a pathway downstream of the *PXY* receptor kinase [9] so we hypothesised that these genes up-regulated in *pxy* should also be up-regulated in *wox4* mutants. qRT-PCR analysis of expression of the same *ERF*'s upregulated in *pxy* mutants also demonstrated increases in expression in *wox4* (Figure 1B). These observations suggest that *ERF* expression is suppressed by the *pxy* signalling pathway and that repression of *ERF* expression occurs downstream of *WOX4*.

**Figure 2:**
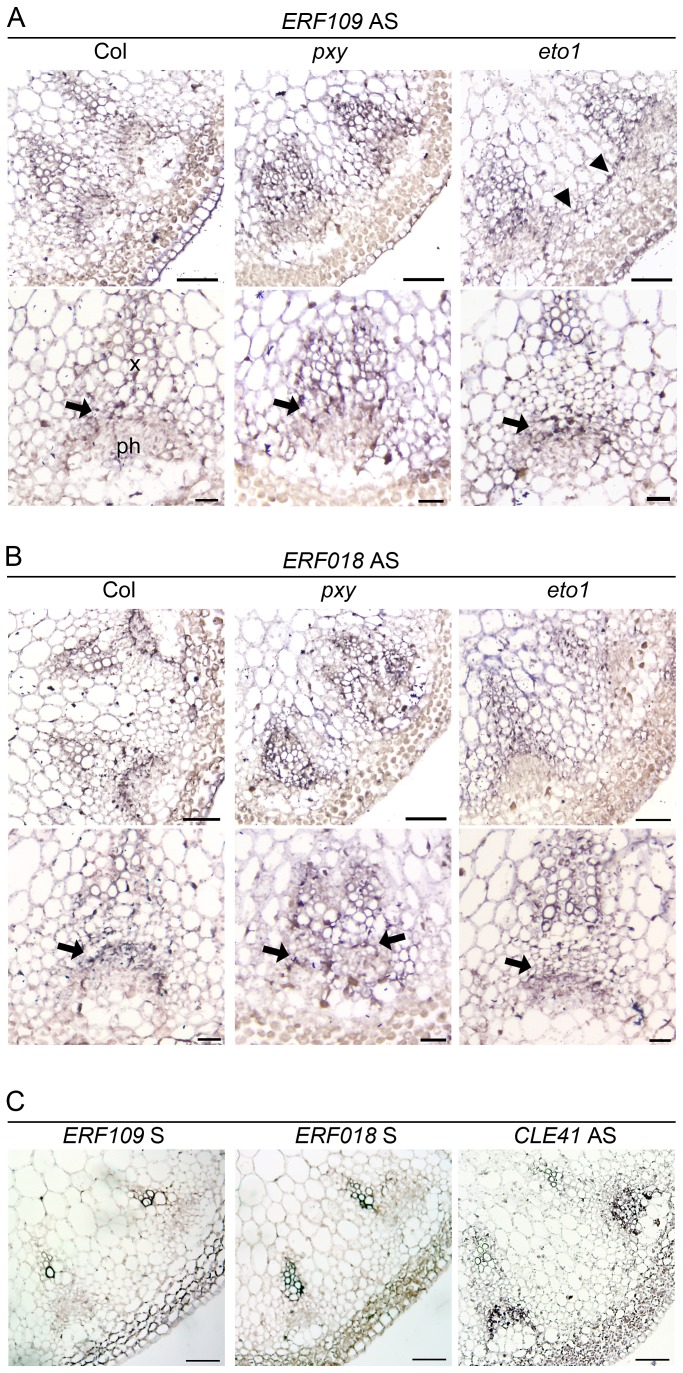
*ERF109* and *ERF018* expression determined by *in situ* hybridization in wild type, *pxy*, and *eto1*. (A) Inflorescence stem *ERF109* expression 4 cm below the shoot apex. Expression appears strongest in the vascular tissue division zone (arrows) in the three genotypes tested but absent from the phloem. Interfascicular *ERF109* expression was apparent in *eto1* mutants (arrowheads; right hand panel). Scales are 50 µm (upper panels) and 25 µm (lower panels). (B) *ERF018* expression is similar to that observed for *ERF109* (arrows show prominent expression in the procambium). Scales are 50 µm (upper panels) and 25 µm (lower panels). (C) Col wild type sections probed with *ERF109* and *ERF018* sense negative controls and *CLE41* antisense positive control. Scales are 50 µm.

We tested for vascular gene expression of two ERF transcription factors, *ERF109* and *ERF018*, using *in situ* hybridization on sections of inflorescence stem from 5 week old plants 4 cm below the shoot apex (Figure 2) and found that Digoxigenin labelled antisense probes labelled many cell types. However, *ERF109* and *ERF018* expression was strongest in vascular bundles. Notably, in wild type, expression for both genes was most prominent in the procambium (arrows in Figure 2A, 2B) but absent from the phloem. In *pxy* mutant vascular tissue, *ERF109* and *ERF018* expression also appeared most prominently in the procambium and xylem (Figure 2). Sense negative controls for both genes did not label tissue above background levels but an antisense *CLE41* positive control specifically labelled phloem tissue (Figure 2C) as previously reported [7]. Quantitative data from microarrays and qRT-PCR, combined with prominent vascular expression of these genes consequently suggests a role for *ERF109* and *ERF018* in vascular tissue.

### *erf* mutants have fewer cells in vascular tissue

**Figure 3:**
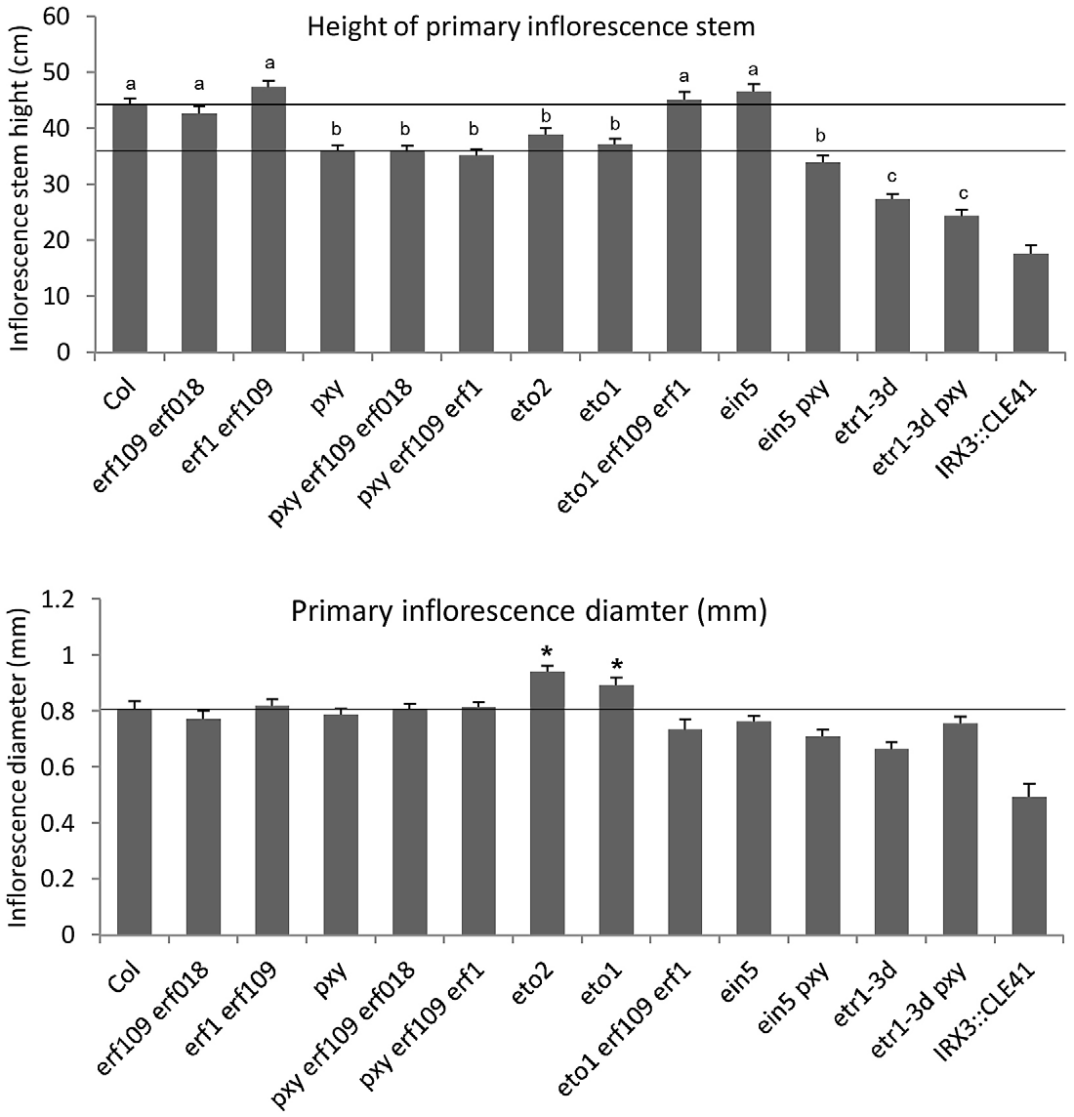
Morphology of plant lines used in this study. Upper graph shows height of plants at 10 weeks. Phenotype of *pxy erf109 erf018*, *pxy erf109 erf1* and *pxy ein5* vascular tissue is not due to a general growth defect as plants were indistinguishable from *pxy* single mutants. Similarly, *etr1-3d* and *pxy etr1-3d* lines were also indistinguishable. Bars marked (a) are similar to wild type; (b) is similar to *pxy*; (c) is similar to *etr1-3d*. Lower graph shows that inflorescence stem diameter is unchanged plant lines used in this study, except for *eto* lines which undergo secondary growth and are consequently larger than wild type (*p*<0.05). *IRX3::CLE41* has more vascular cells than wild type but is of reduced height and stem diameter demonstrating that there is not a simple correlation between vascular cell division and overall plant morphology.

**Figure 4:**
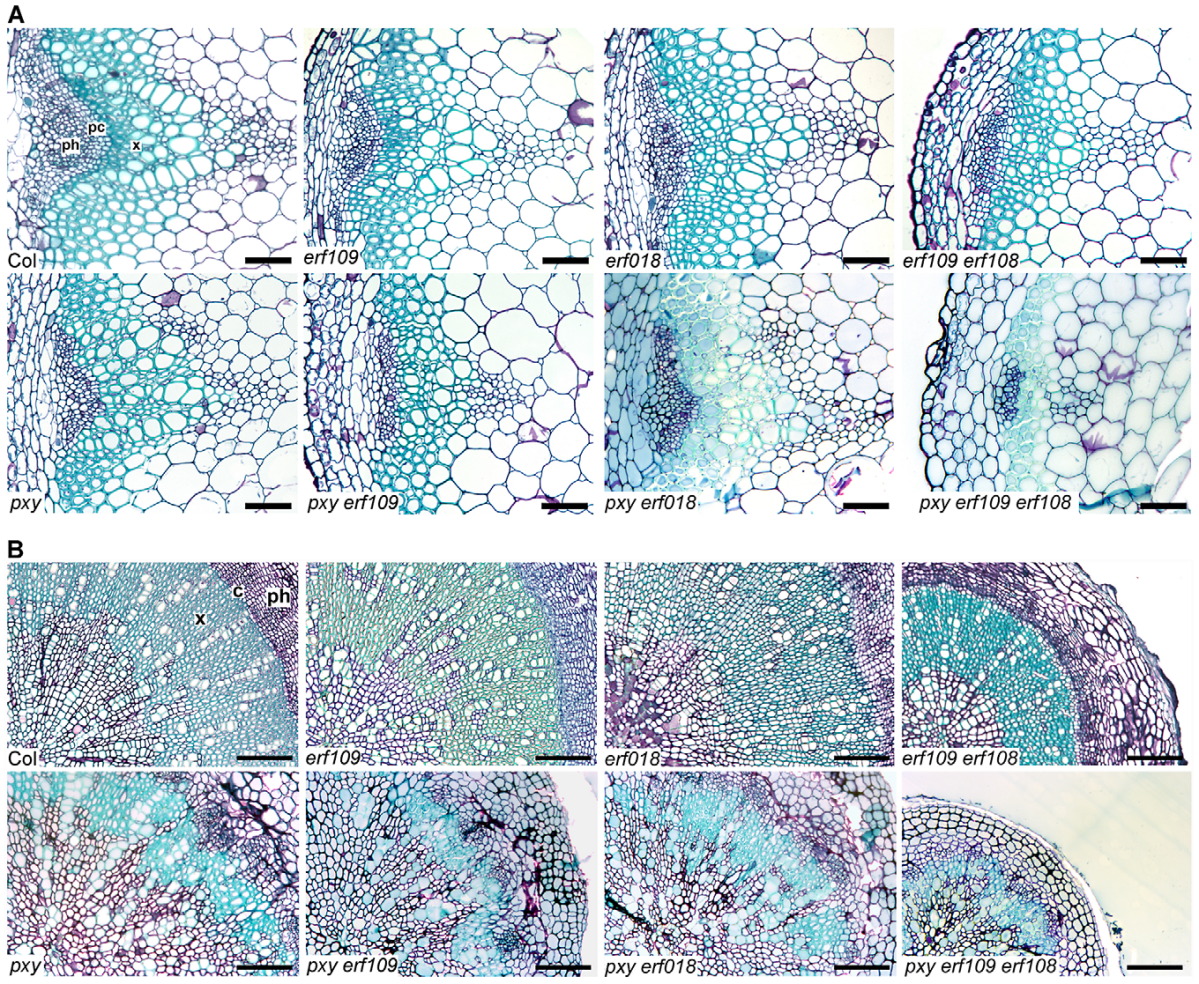
*pxy* interacts with *erf* transcription factors. (A) Combinations of *erf109*, *erf018* and *pxy* mutants. In comparison to wild type, *erf109*, *erf018* and *pxy* vascular bundles have similar numbers of cells to wild type. *erf109 erf018*, *pxy erf109* and *pxy erf109 erf018* vascular tissue demonstrates a reduction in size compared to single mutants. Vascular bundles are from the base of 10 week inflorescence stems; scales are 50 µm. (B) *erf109 erf018* hypocotyls are reduced in size compared to single mutant and wild type counterparts. *pxy erf109 erf018* hypocotyls are smaller than those of parental lines. Images are from 10 week old hypocotyls; Scale bars are 100 µm. x is xylem, pc is procambium, c is cambium, ph is phloem.

**Figure 5:**
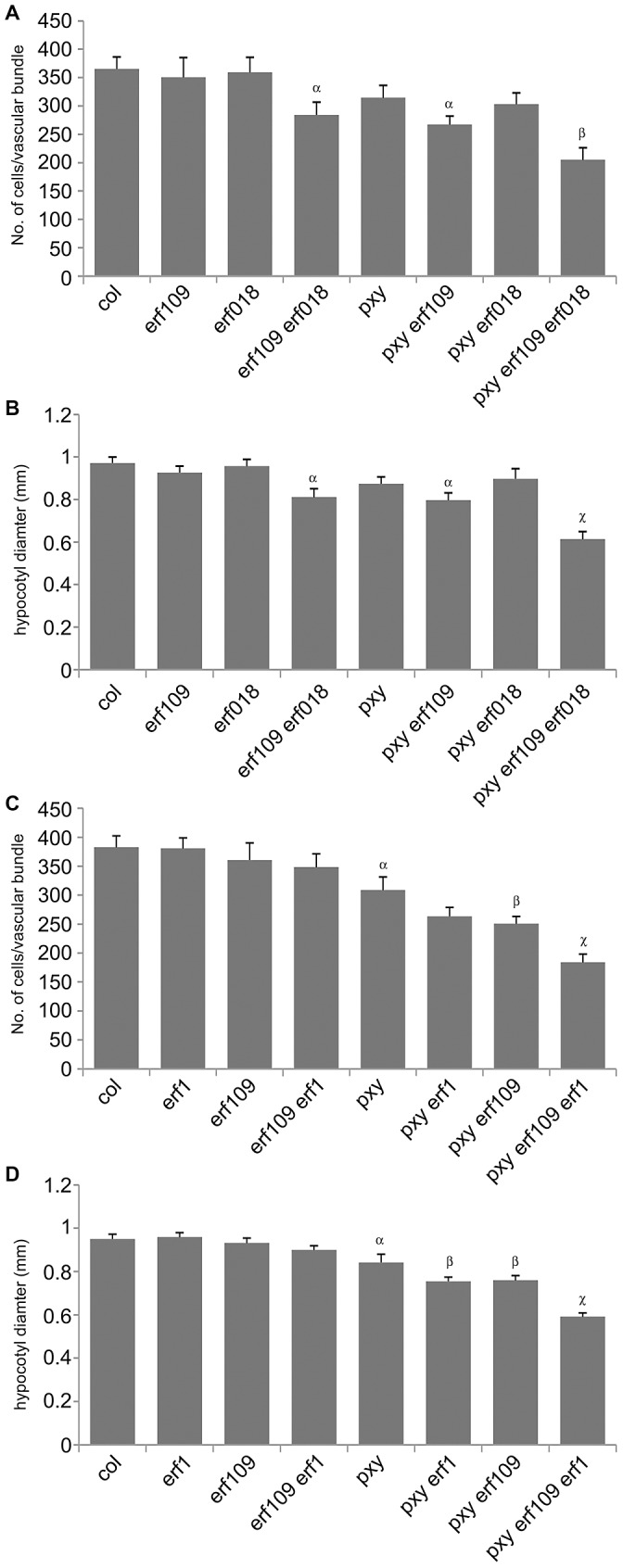
Quantitative analysis of vascular cell division in *erf109*, *erf018*, *erf1*, and *pxy* mutant combinations. (A) and (B) *erf109*, *erf018* and *pxy* mutant combinations. (A) Cells per vascular bundle at the base of the inflorescence stem of 10 week plants. (B) Hypocotyl diameter (mm) of plants at 10 weeks. (C) and (D) *erf109*, *erf1* and *pxy* mutant combinations. (C) Cells per vascular bundle at the base of the inflorescence stem of 10 week plants. (D) Hypocotyl diameter (mm) of plants at 10 weeks. α is significantly smaller than Col; β is significantly smaller than *pxy*; χ is significantly smaller than respective double mutants. *p*<0.05. Error bars are standard error.

To determine the functional relevance of the gene expression changes observed in *ERF*'s, we identified *erf018* and *erf109* loss-of-function mutants in publicly available T-DNA insertion libraries as these genes demonstrated relatively large increases in expression in *pxy* mutants. A confirmed T-DNA insertion within the coding sequence of *ERF109* (Salk_150614) was renamed *erf109-1*, however, no insertion mutant was available that disrupted the coding sequence of *ERF018*. Salk_109440 line (*erf018-1*) was found to harbour a T-DNA insertion 142 base pairs upstream of the transcriptional start site and 249 base pairs upstream of the ATG. qPCR was used to analyse the expression of *ERF018* in these lines and we found that expression was reduced to 60% of wild type levels (Figure S2) indicating that *erf018-1* is a weak allele. Gross morphology of *erf018*, *erf109* single and *erf109 erf018* double mutants appeared identical to wild type counterparts (Figure 3) and the number of cells in *erf018* and *erf109* mutant vascular bundles was unchanged from wild type in 10 week old inflorescence stems (Figure 4A, Figure 5A). In contrast, *erf109 erf018* double mutants demonstrated a small but significant reduction in the number of cells per vascular bundle (78% of wild type; Figure 4A, Figure 5A) suggesting that *ERF109* and *ERF018* act redundantly in promoting cell division in vascular bundles.

The role of *ERF109* and *ERF018* in secondary growth was addressed in *Arabidopsis* hypocotyls. Several authors have used hypocotyl diameter as a measure of cell division during secondary growth [3], [7], [8], [19], [20], [21]. Consistent with our observation that *erf109 erf018* lines had reductions in vascular cell division in stems, hypocotyl diameter was also reduced in *erf109 erf018* double mutants to 83% of wild type diameter (Figure 4B, Figure 5B). Consequently, *ERF109* and *ERF018* are required for promotion of vascular cell division during both primary and secondary growth.

### *ERF*s act in a parallel pathway to PXY signalling

It was clear from our analysis that *ERF109* and *ERF018* are required for promoting vascular cell divisions. Since these transcription factors are upregulated in *pxy* mutants it may be hypothesised that they represent a mechanism by which vascular cell division is maintained in the absence of *PXY*. *pxy erf109 erf018* triple mutants were therefore generated with the expectation that if *ERF* transcription factors do compensate for loss of *pxy* then *pxy erf109 erf018* lines would demonstrate a significant reduction in cell number when compared to *pxy*, *erf109 erf018* or wild type. *pxy* mutant vascular bundles have been previously characterised with intercalated xylem and phloem, however, in inflorescence stems of 5 week old plants no differences in vascular cell number were observed [2]. We reasoned that differences in the number of cells in *pxy* vasculature may be observed in 10 week old tissue, particularly in hypocotyls which undergo continuous radial expansion, as subtle differences in the rate of cell division would have time to accumulate. All experiments on 10 week old tissue in this manuscript (see below) demonstrated a trend towards a reduction in cell number in *pxy* mutant vascular bundles compared to wild type (see below), however, in this instance, differences proved not to be statistically significant (Figure 4A, Figure 5A). Consistent with our hypothesis, vascular bundles *of pxy erf109* mutants demonstrated a 27% reduction in cell number compared to wild type in contrast with *pxy* and *erf109* which showed no significant difference (Figure 4A, Figure 5A). Consequently, clear defects in *pxy* mutant vascular cell number only became apparent when *pxy* was combined with an *erf109* mutant. *pxy erf018* and *pxy erf018 erf109* were also generated to determine whether *erf018* demonstrated a similar interaction with *pxy. pxy erf018* double mutant inflorescence stem vascular tissue did not differ from parental lines (Figure 4A, Figure 5A), however, *pxy erf018 erf109* lines demonstrated a 44% reduction in cells/vascular bundle demonstrating a significant enhancement of the *pxy erf109* phenotype (Figure 4A, Figure 5A).

When analysing secondary growth in *pxy erf109* double mutant hypocotyls, we found that the relationship between *erf109* and *pxy* was similar to that observed in vascular bundles. *pxy erf109* hypocotyls had the characteristic altered orientation of cell division associated with *pxy* mutants [7] but the hypocotyl diameters were narrower than either *pxy* or *erf109* single mutants (Figure 4B). The decrease in hypocotyl diameter was most dramatic in *pxy erf109 erf018* mutants where hypocotyl diameters were only 63% of that observed in wild type. These observations are consistent with fewer cell divisions having occurred in the triple mutant than the respective doubles, single mutants and wild type (Figure 4B, Figure 5B). As with our observation in vascular bundles, defects in vascular cell number are greatly enhanced when *pxy* mutants are combined with mutations in the *ERF* transcription factors *erf018* and *erf109*.

We further examined *ERF* function in PXY signalling by analysing the function of *AtERF1* (At1g17500; upregulated in both *pxy* and *wox4* mutants; Figure 1B, Table S1). A T-DNA mutant (Salk_036267) was isolated and used to test whether *AtERF1* acted similarly to *ERF018* and *ERF109*. *erf1* single and *erf1 erf109* double mutants were indistinguishable from wild type, and although *erf1 pxy* double mutants were suggestive of a reduction in the size of vascular bundles compared to *pxy* single mutants, differences proved not significant (Figure 5C). In contrast, *pxy erf1 erf109* triple mutants demonstrated a dramatic decrease in the number of cells/vascular bundle (48% of that observed in wild type), a significant reduction when compared to respective single and double mutants when assayed at the base of the inflorescence stems of 10 week old plants (Figure 5C). Similarly, *pxy erf109 erf1* lines demonstrated reduced hypocotyl diameter (52% of wild type) when compared to control lines (≤80% of wild type; Figure 5D). *erf1* therefore enhances vascular cell division defects of *erf109 pxy* mutants in both inflorescence and hypocotyl. These data are consistent with a role for *AtERF1*, *ERF109* and *ERF018* in promoting vascular cell division in the absence of *PXY*.

### *ERF* expression in the context of ethylene signalling

**Figure 6:**
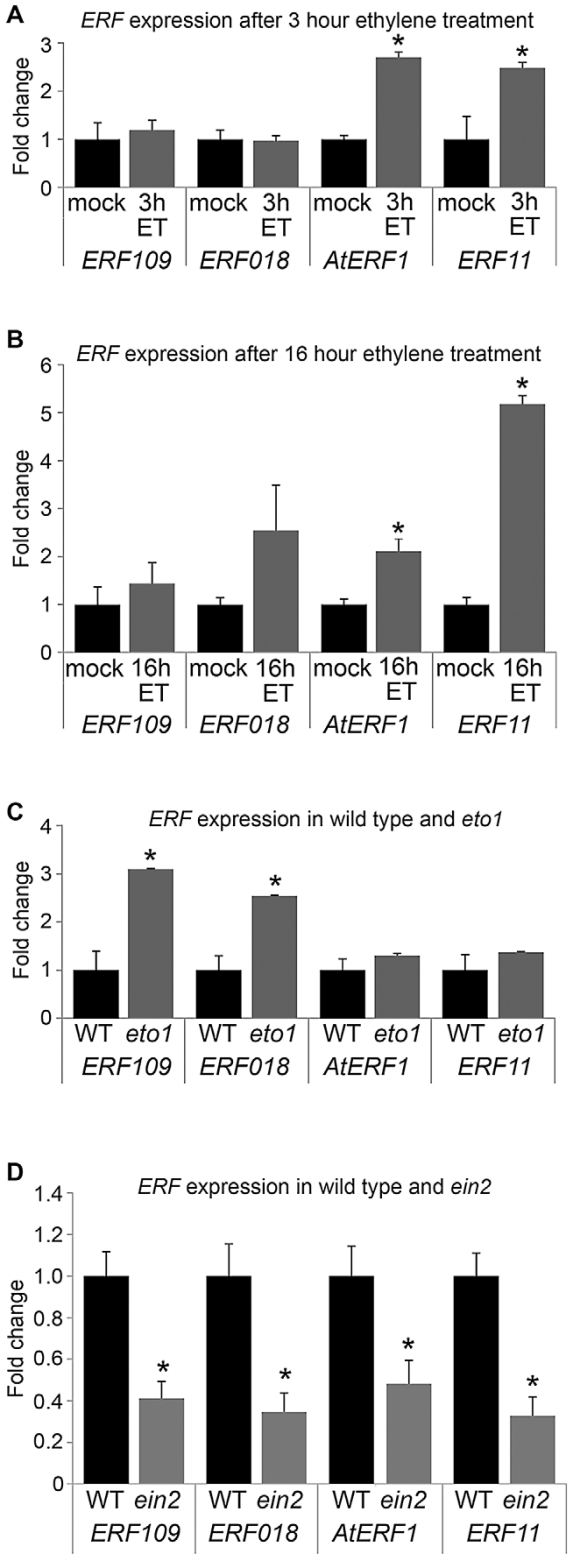
*ERF* expression responds to ethylene. (A) qRT-PCR showing *ERF* expression in inflorescence stems in response to 3 hour ethylene exposure (grey bars) compared to mock treatment (black bars). (B) qRT-PCR showing *ERF* expression in inflorescence stems in response to 16 hour ethylene exposure (grey bars) compared to mock treatment (black bars). (C) Inflorescence stem *ERF* expression in plants that over-produce ethylene (*eto1*; grey bars) compared to wild type (black bars). (D) *ERF* expression in *ein2* mutant inflorescence stems (grey bars) compared to wild type (black bars). *Expression significantly different from wild type controls (*p*<0.05). Samples were measured in technical triplicates on biological triplicates.

Five of the *ERF* genes upregulated in *pxy*; *AtERF1*, *ERF2*, *ERF5*, *ERF003*/*Atg525190* and *ERF11* have previously been shown to be induced by ethylene [22], [23], [24], [25]. Furthermore, an enzyme responsible for catalysing the rate-limiting step of ethylene biosynthesis, *ACS6* (At4g11280) was upregulated 2.5 fold in *pxy* mutants (Table S1; Figure 1B). Consequently, we hypothesised that the increase in expression of *ERF* transcription factors in *pxy* and *wox4* mutants may be the result of an increase in ethylene signalling. To determine whether these genes also demonstrated elevated expression in stems of plants with higher levels of ethylene than wild type, their response to ethylene exposure was tested. We subjected five week old wild type *Arabidopsis* plants to ethylene stimuli of 3 hours, 16 hours and also made use of *ethylene overproducer1* (*eto1*) mutants which produce more ethylene than wild type [26]. Expression levels of *ERF's* were compared in inflorescence stems using qRT-PCR (Figure 6). Expression was increased in response to exogenous ethylene treatment as in plants exposed to ethylene for 3 hours, *AtERF1* and *ERF11* underwent a 3-fold induction (Figure 6A) and following a 16 hour treatment 2- and 5-fold inductions were observed (Figure 6B). Increased *ERF109* and *ERF018* expression of approximately 3-fold was observed in *eto1*. Consequently *ERF109*, *ERF018*, *AtERF1* and *ERF11* are ethylene responsive; however, the dynamics of induction varies in inflorescence stems. *ERF1* and *ERF11* demonstrated an early ethylene response and *ERF018* and *ERF109* expression was increased in response to a constitutive ethylene production (Figure 6).

To confirm the relationship between *ERF* expression and ethylene in stems, we carried out the converse experiment. *ERF* levels were determined in *ethylene insensitive 2* (*ein2*) plants in which the ethylene signal transduction pathway is thought to be entirely abolished [27]. Consistent with *ERF109*, *ERF018*, *AtERF1* and *ERF11* acting downstream of the ethylene response in inflorescence stems, expression of the genes tested was reduced by half (Figure 6D). It is notable that *ein2* mutants do not demonstrate reductions in vascular cell number (see below). Consequently, differences in *ERF* expression cannot be explained by phenotypic differences in vascular tissue and are likely the result of reduced ethylene signalling.

### Ethylene promotes radial growth in *Arabidopsis*

**Figure 7:**
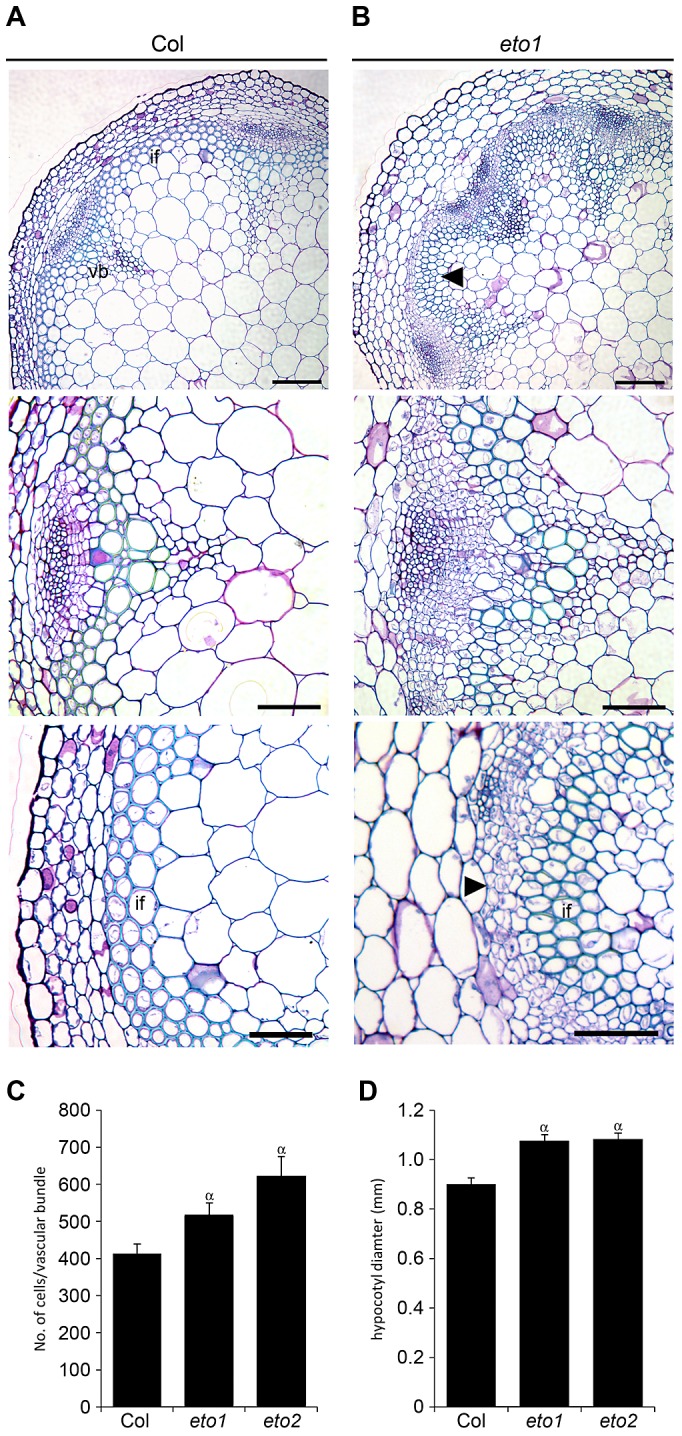
Increased vascular cell divisions in *eto1* and *eto2* mutants. (A) Transverse sections of toluidine blue stained wild type 5 week inflorescence stems. (B) *eto1* mutants inflorescence stems have more procambium than wild type (compare middle panels), and initiate secondary growth between vascular bundles at an early age (arrowheads in upper and lower panels). (A and B) Scales are 100 µm (upper), or 50 µm (middle and lower panels). vb is vascular bundle; if interfascicular region. (C) Graph showing cells per vascular bundle at the base of the inflorescence stem of 10 week Col, *eto1* and *eto2* plants. (D) Graph showing hypocotyl diameter (mm) of Col, *eto1* and *eto2* plants at 10 weeks. (C and D) α is significantly larger than Col (*p*<0.05); error bars are standard error.

Reports in poplar have demonstrated that ethylene promotes vascular cell division during secondary growth [15], so in order to determine whether ethylene, and therefore *ERF*'s, function similarly in *Arabidopsis* we analysed the inflorescence stems of *eto1* mutants at six weeks and found that they exhibited an increase in the number of procambial cells (Figure 7A–7B). *eto1* mutants also demonstrated early onset of secondary growth as vascular cell divisions were observed in the interfascicular region prior to any divisions in wild type plants at an equivalent stage of development (Figure 7A–7B). This phenotype was particularly evident when *eto1* mutant vascular sections were subjected to *in situ* hybridization with *ERF109* antisense probes. In wild-type, labelling was absent from interfascicular tissue but present in *eto1* (Figure 2). The phenotypic consequences of constitutive ethylene production were confirmed by analysis of *eto2* mutants [28]. At ten weeks, as with *eto1* mutants, *eto2* plants had larger vascular bundles and cell divisions between vascular bundles indicative of secondary growth which were absent in wild type (Figure S3). To confirm that the observed differences in *eto1* and *eto2* were significant, the number of cells in vascular bundles of 10 week inflorescence stems and hypocotyl diameters were determined. Increases in vascular cell number of 21% for *eto1* and 34% for *eto2* in inflorescence stems and increases in hypocotyl diameter of 19% and 31%, respectively were apparent (Figure 7C–7D). Our data are therefore consistent with the idea that elevated levels of ethylene result in both an increase in vascular cell division and increased expression of *ERF* transcription factors which we have shown are required to promote vascular cell division in the absence of *PXY*.

**Figure 8:**
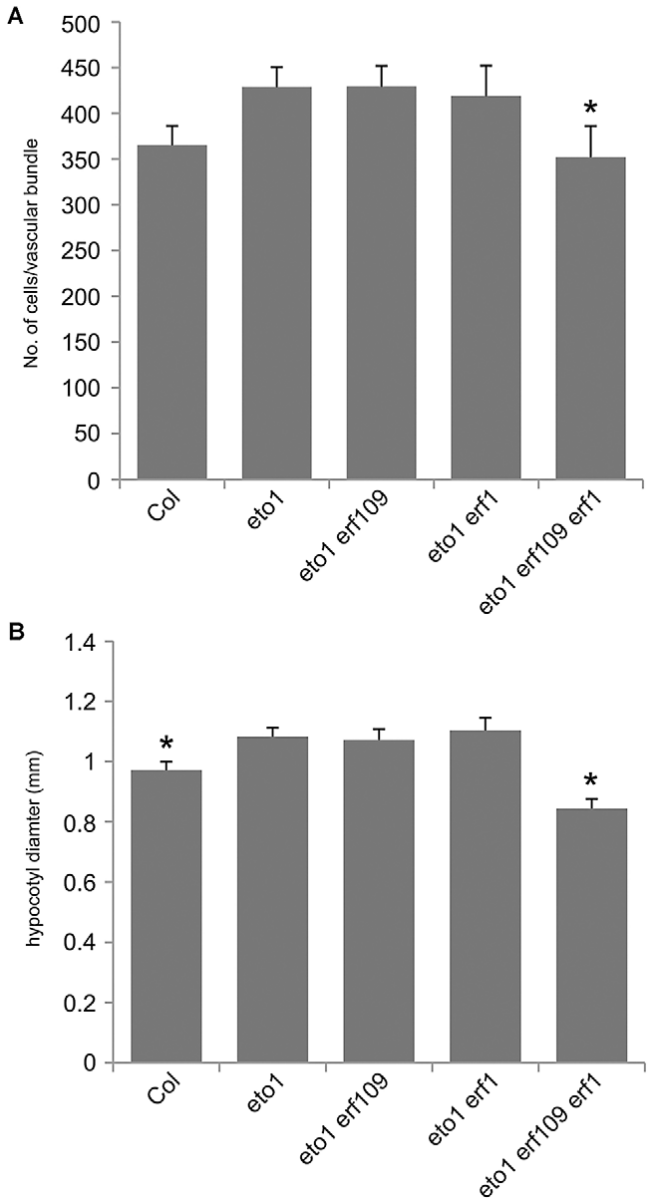
*ERF* genes are required for the *eto1* vascular phenotype. (A) Graph showing cells per vascular bundle at the base of the inflorescence stem of 10 week Col, *eto1*, *eto1 erf109*, *eto1 erf1* and *eto1 erf109 erf1* plants. *eto1 erf109 erf1* plants are significantly smaller than *eto1* single mutants. (B) Graph showing hypocotyl diameter (mm) of Col, *eto1 erf109*, *eto1 erf1* and *eto1 erf109 erf1* plants at 10 weeks. *ERF109* and *ERF1* are required for increases in hypocotyl diameter observed in *eto1*. (A and B) * significantly smaller than *eto1* (*p*<0.05); error bars are standard error.

To confirm that *ERF* transcription factors upregulated in *pxy* mutants were required for vascular *eto* phenotypes and therefore ethylene mediated vascular expansion, we generated *eto1 erf109 erf1* triple mutants. *eto1 erf109* and *eto1 erf1* double mutant lines were indistinguishable from *eto1* single mutants, but in *eto1 erf109 erf1* lines, vascular cell number was significantly smaller than that observed in *eto1* (Figure 8). Furthermore interfascicular cell divisions that were sometimes present in *eto1* lines were not observed in *eto1 erf109 erf1* counterparts (Figure S4), and *eto1* stems demonstrated an increase in diameter compared to those of *eto1 erf109 erf1* (Figure 3), consistent with a requirement for *ERF*'s in *eto* secondary growth phenotypes. Consequently, we have demonstrated that the *ERF* transcription factors that demonstrate increased expression in *pxy* mutants are upregulated in response to ethylene, their expression is reduced in ethylene signalling mutants and they are required for the phenotypic consequences of ethylene over-production in vascular tissue.

### Crosstalk between ethylene and *pxy* signalling

**Figure 9:**
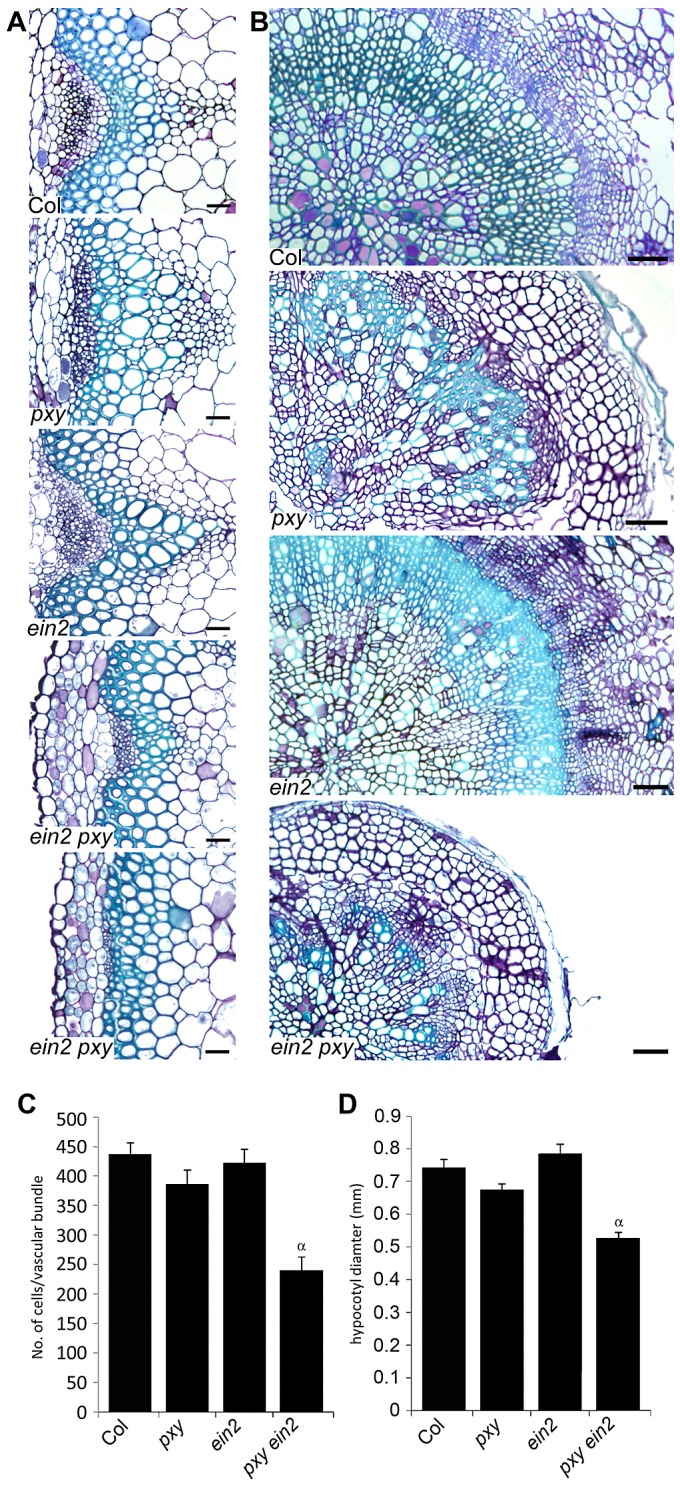
Analysis of vascular cell division in *ein2* and *pxy* mutant combinations. (A) Combinations of *pxy* and *ein2* inflorescence stems. *ein2* is similar to wild type. *pxy ein2* is smaller than *pxy* and *ein2* lines. In extreme cases (lower panel), *pxy ein2* vascular bundles are extremely small. Scale bars are 25 µm. (B) Transverse sections through hypocotyls show that compared to wild type, *pxy* mutants have disrupted organisation due to loss of orientation of cell division. *ein2* is indistinguishable from wild type. *pxy ein2* hypocotyls are severely reduced in size and lack organisation like *pxy* mutants. Scale bars are 50 µm. (C) Cells per vascular bundle at the base of the inflorescence stem of 10 week plants. (D) Hypocotyl diameter (mm) of plants at 10 weeks. (C and D) α is significantly smaller than Col (*p*<0.0001); error bars are standard error.

To directly address the relationship between PXY and ethylene signalling, we crossed mutants that are unable to respond to ethylene to *pxy*. *ein2* encodes an integral membrane of unknown function that is essential for ethylene signal transduction [27] and is the only single mutant thought to entirely abolish ethylene signalling [27]. *pxy ein2* double mutants developed normal rosettes and inflorescence stems were initiated normally, however, the plants senesced early so analysis of ten week plants, consistent with quantitative phenotypic analysis elsewhere in this manuscript was not possible. Analysis was carried out on six week old plants but at this developmental stage, wild type plants had similar numbers of cells in vascular bundles as present at ten weeks suggesting that vascular proliferation in the stem was complete (Figure 9A, 9C). Wild-type and *ein2* vasculature in inflorescence stems were indistinguishable, with no significant difference in vascular cell number. *pxy ein2* mutant vascular tissue demonstrated a dramatic reduction in vascular cell number (55% of wild type), having significantly fewer cells than *pxy* or *ein2* single mutants (Figure 9A, 9C) and clearly demonstrating that *ein2* is required for maintenance of vascular tissue in *pxy* mutants. Similar results were observed in the hypocotyl (Figure 9B, 9D) with *pxy ein2* lines significantly smaller than *ein2* or *pxy* single mutants.

To confirm the results obtained with *ein2*, two further mutants in the ethylene signal transduction pathway were analysed. *ethylene receptor1* (*etr1*) and *ethylene insensitive5* (*ein5*) encode an ethylene receptor [29], and an exoribonuclease involved in ethylene signalling [30], [31], respectively. Neither *etr1-3d* nor *ein5-1* exhibit the triple response and are partially ethylene insensitive [32]. In primary vascular tissue in inflorescence stems, in common with *ein2* mutants, *ein5* and *etr1-3d* were indistinguishable from wild type (Figures S5, S6) but in both cases a dramatic enhancement of the reduction in vascular cell number observed in *pxy* mutants (see above) was observed when *pxy etr1-3d* double mutants were analysed (Figures S5, S6).

Analysis of the role of *etr1-3d* and *ein5* in hypocotyl secondary growth was also carried out. Hypocotyl diameters were measured at 10 weeks and *ein5* was found not to differ from wild type, however *etr1-3d* demonstrated a small reduction (Figure S6). Although this differed from observations in *ein2* and *ein5*, this is likely due to the age of plants tested with respect to *ein2* and differences in the level of reduction of ethylene signalling with respect to *ein5*. In common with *ein2*, *etr1-3d* and *ein5* both strengthened the *pxy* phenotype as double mutants were smaller than respective singles (Figures S5, S6).

If *ERF109*, *ERF018* and *ERF1* are targets of an ethylene-induced signalling mechanism that is upregulated in the absence of *pxy*, then *pxy erf* mutants should appear similar to those of *pxy ein2*, *pxy etr1-3d* and *pxy ein5*. As such, *pxy erf109 erf018* and *pxy erf109 erf1* vascular tissue was similar to that of *pxy ein5*, *pxy etr1-3d* and *pxy ein2* as in all instances, the *pxy* cell division phenotype was enhanced. It is notable that ethylene signalling does not appear to greatly influence PXY signalling. Expression levels of *CLE41*, *CLE42*, *PXY* and *WOX4* were unchanged in *ein2* mutants or *erf109 erf018* plants (Figure S7). *CLE41*, *CLE42* and *WOX4* expression was also unchanged in plants exposed to an ethylene stimulus, however, ethylene did promote *PXY* expression (Figure S7) suggesting that *PXY* is to some extent ethylene responsive.

## Discussion

Up regulation of the PXY signal transduction pathway by over expression of the CLE41 ligand results in massively increased vascular cell divisions, however, *pxy* mutants exhibit only limited reductions in cell division. We have identified a group of 12 *ERF* transcription factors that are upregulated in *pxy* mutants (Table S1; Figure 1). Loss of function analysis of three of these genes; *ERF109*, *ERF018* and *AtERF1* resulted in plants with inflorescence stems that were characterised by reduced numbers of vascular cells suggesting that these genes promote cell division in vascular meristems (Figure 4, Figure 5). This data suggests that these *ERF transcription factors* form part of a mechanism that is up-regulated in response to loss of *pxy*.

**Figure 10:**
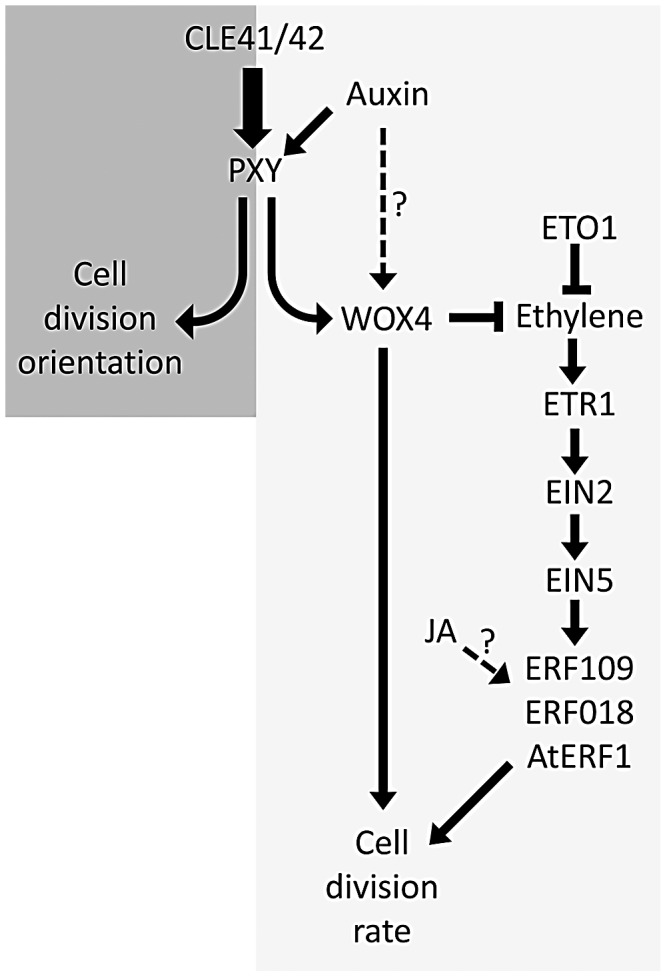
Model showing ethylene and PXY signalling act in parallel pathways in vascular development. Crosstalk between these two signalling pathways contributes to levels of ERF transcription factors and control of the number of vascular cell divisions. Auxin and JA also influence this network.

Previous authors have demonstrated that five of the genes identified have increased expression in response to ethylene in seedlings [22], [23], [24], [25]. We have demonstrated that several of the family members are upregulated in stems of ethylene overproducing *eto1* mutants or in plants subjected to ethylene treatment (Figure 6). Furthermore these *ERF*'s are required for the increased vascular tissue observed in *eto1* plants (Figure 7, Figure 8). An involvement of ethylene in vascular cell division in *pxy* plants is supported by analysis of *ein2*, *ein5* and *etr1-3d* mutants. *EIN2*, *EIN5* and *ETR1* are required for normal ethylene signal transduction [32] and *pxy ein2*, *pxy ein5* and *pxy etr1-3d* plants had significant reductions in the number of vascular cells compared to single mutants or wild type (Figure 9; Figures S5, S6). Taken together, our results demonstrate that *ERF* transcription factors promote vascular cell division, that their expression is influenced by PXY-repression of ethylene signalling, and consequently, these signalling pathways interact to control the rate of cell division in plant vascular tissue (Figure 10).

1-aminocyclopropane-1-carboxylic acid synthase 6 (*AtACS6*) is also upregulated in *pxy* (Table S1; Figure 1B). ACS enzymes catalyse the rate-limiting step of ethylene biosynthesis [33], [34], i.e. conversion of *S*-adenosylmethionine to ACC [35]. Ethylene has previously been shown to promote cell division in the organising centre of *Arabidopsis* roots [36], and in the cambium of hybrid poplar [15]. In tree species, ethylene is produced in association with physical stress [13], and is known to have a role in promoting development of tension wood [15]. Our results suggest that it may have a more general role in regulating the rate of cell division in the cambium (Figure 10), particularly as *etr1-3d* mutants demonstrated significant reductions in radial growth compared to wild type in hypocotyls (Figure S6). These conclusions are also consistent with earlier studies that demonstrate that in trees ethylene levels are higher at the height of the growing season than towards the end [13].

It may be case that *ERF109*, *ERF018*, *AtERF1* and other *ERF*'s upregulated in *pxy* are essential components in the vascular developmental programme and their expression can be modulated by ethylene and other external factors. *ERF* transcription factors affect a variety of processes [17], , but strikingly, *ERF018* and *AtERF1* have also been described as being up-regulated by jasmonic acid [18], [25]. Jasmonic acid has recently emerged as a key modulator of cell division in the cambium [39] and consequently *ERF018* and *AtERF1* may be key integration points for vascular development. In support of this hypothesis (Figure 10), phenotypes were observed in *erf018* mutants (in combination with *erf109*) despite the weak nature of the *erf018* allele identified in this study. Our observations, and those of previous authors are consistent with an emerging picture that many of these transcription factors form part of a network [40] and how the plant responds to them is very much context dependent [41]. The *ERF* transcription factors analysed in this study have been suggested as having roles unrelated to vascular development [17], [18], [42], [43] and consequently, *ERF109* and *ERF018* have broad expression patterns (but are nevertheless expressed in vascular tissue), however, single mutants and neither *erf109 erf1* nor *erf109 erf018* double mutants demonstrated visible architectural defects, such as a change in height (Figure 3), suggesting that reductions in vascular cell number in these lines is not the consequence of a general disruption to plant growth.

The phytohormones brassinosteroid [44], cytokinin [45], [46], [47], strigolactone [48] and auxin [49], [50] have also been shown to have roles in *Arabidopsis* vascular development, and have been shown to be regulate each other's biosynthetic pathways [25]. Brassinosteroid upregulates genes required for ethylene biosynthesis and auxin up regulates genes involved in cytokinin biosynthesis. More complex interactions occur between ethylene and auxin, brassinosteroid and auxin as well as cytokinin and brassinosteroid, where phytohormone biosynthesis genes are both induced and repressed in response to respective phytohormone treatments [25]. A proper understanding of vascular cell division and differentiation will need to take into account interactions between these signalling pathways and their downstream targets.

One phytohormone in addition to ethylene that has been placed in a network with PXY signalling is auxin. *WOX4* is positively regulated by auxin which has led to the suggestion that auxin lies upstream of PXY signalling in a hypothesis that is supported by the observation that part of the *WOX4* response to auxin is *PXY*-dependent [10]. In support of this hypothesis, *ERF* genes upregulated in *pxy* mutants also demonstrated increased expression in *wox4* lines (Figure 1B). However, *WOX4* regulation may not depend entirely on PXY signalling and could also be regulated by an auxin-dependent, *PXY*-independent mechanism [51] because despite the observation that *PXY* is required for the *WOX4* auxin response, *WOX4* expression nevertheless increases in *pxy* mutants subjected to a 1 day auxin induction [10]. Further evidence for a *WOX4*, *PXY*-independent response comes from the observation that *wox4* enhances *pxy* mutants [9]. If *WOX4* expression was entirely controlled by *PXY* signalling *wox4 pxy* double mutants and respective single mutants would have identical phenotypes. Experiments presented here, and by previous authors therefore place PXY signalling in a network containing auxin, ethylene and JA signalling (Figure 10).

The procambium and cambium are meristematic tissues and as such, demonstrate similarities with the shoot apical meristem (SAM) and root apical meristem (RAM). All three structures require CLAVATA (CLV) -like, and phytohormone signalling mechanisms for their regulation. In the SAM, CLE41-related CLV3 is secreted from stem cells and binds to the PXY-related CLV1 receptor [52], . This precipitates a signal which culminates in negative regulation of *WUSCHEL* (*WUS*), a homeobox gene which promotes stem cell fate – and therefore cells that secrete CLV3. This feedback loop enables the plant to dynamically regulate the size of its apical stem cell population thus balancing organ generation with maintenance of its stem cells [56], [57]. *WUS* expression is also controlled by cytokinin signalling, which is thought to add robustness to the feedback mechanism [58]. It is tempting to speculate that the relationship between PXY and ethylene signalling acts similarly. In this case, interaction between the PXY and ethylene pathways culminates in appropriate regulation of downstream transcription factors required to regulate the rate of cell division and recruitment of daughter cells into xylem and phloem.

## Materials and Methods

### Generation of plant stocks

Plant growth conditions, and *pxy* alleles have been described previously [2]. T-DNA insertion lines in *ERF018* (salk_109440), *ERF109* (salk_150614), *AtERF1* (salk_036267) and *wox4-1* (GABI-Kat_462G01) were identified using the TAIR database [59] and confirmed using PCR. Insertion lines and *eto1-1*, *ein5-1*, and *etr1-3d* were obtained from NASC. *erf109 erf018*, *pxy erf109*, *pxy erf018*, *pxy erf018 erf109*, *erf1 erf109*, *pxy erf1* and *pxy erf1 erf109* lines were identified in segregating F2 populations. Primers for *pxy* genotyping have been described previously [7]. Oligos SALK_ERF109LB and SALK_ERF109RB (CGCGATGCTTTGTAGGAGTAG and TGTCAGGGTTTTTCCAGTGAC), SALK_ERF018LB and SALK_ERF018RB (TTCATGCTCATGATGATGAGC and ATCGACGGTGGATTATTAGGG) and salk-ERF1-F and salk-ERF1-R (CGTTCCTAACCAAACCCTAGC and TCCTACTCTTCTCCCTGCTCC) were used for the identification of *erf109*, *erf018* and *erf1* mutants. *pxy ein2*, *pxy ein5* and *pxy etr1-3d* doubles were selected in the F3 generation from families that were ethylene insensitive as determined using a triple response screen [26].

Ethylene treatments of plants prior to measurement of *ERF* expression in inflorescence stems was carried out by placing *Arabidopsis* plants in a sealed container and generating ethylene gas to a maximal concentration of 500 µl l^−1^ of ethylene gas as described previously [60].

### Gene expression analysis

For comparison of wild type and *pxy* transcriptomes, Col-0 and *pxy-3* lines were used. For each replicate, plants were germinated on MS agar plates prior to transfer to soil (6 plants per 10 cm pot), where they were grown on for 5 weeks under long day (16/8 h light/dark) conditions at which point the inflorescence stem had 4–6 expanded siliques. Pots were randomised and rotated daily. For each replicate, the 6 primary inflorescence stems were taken from all the plants in a pot. Cauline leaves and side branches were removed. Stems were divided into 4 sections of equal size and RNA was isolated from the third section from the top using TRIzol Reagent (Invitrogen). RNA was sent to the University of Manchester Genomic Technologies Facility (http://www.ls.manchester.ac.uk/research/facilities/microarray/) where it was assessed for quality. ATH1 Affymetrix GeneChip oligonucleotide arrays were used to analyse the gene expression from each sample. Biotinylated cDNA samples from three biological replicates of *pxy* and wild type stems were synthesised and hybridized to Arabidopsis ATH1 genome oligonucleotide arrays. Technical quality control was performed with dChip (V2005; www.dchip.org), using the default settings [61]. Background correction, quantile normalization, and gene expression analysis were performed using RMA in Bioconductor [62]. Differential expression analysis was performed using Limma using the functions lmFit and eBayes [63]. Microarray data has been submitted in a MIAME compliant standard to the Array Express database (Experiment E-MEXP-2420, http://www.ebi.ac.uk/arrayexpress).

For RT-PCR, RNA was isolated using Trizol (Invitrogen). cDNA synthesis, following DNase treatment, was performed using Superscript III reverse transcriptase (Invitrogen). All samples were measured in technical triplicates on biological triplicates. The qRT-PCR reaction was performed using SYBR Green JumpStart Taq ReadyMix (Sigma) using an ABI Prism 7000 machine (Applied Biosystems) with the standard sybr green detection programme. A melting curve was produced at the end of every experiment to ensure that only single products were formed. Gene expression was determined using a version of the comparative threshold cycle (Ct) method. The average amplification efficiency of each target was determined using LinReg [64] and samples were normalised to *18S rRNA* or *ACT2*. Primers for qRT-PCR are described in Table S2.

*ERF109* and *ERF018* probe templates for Digoxigenin-labelling of mRNA were generated by PCR amplification and subsequent cloning of products into pENTR-D-topo using primers (caccaacagagtcgcaaga and catgctttcttgttcttgttc for *ERF109*; caccaattcaaccaaaccgaat and ccagatttctccatgactcca for *ERF018*). The resulting plasmids were used with M13 forward and reverse primers to generate a template for antisense probes, and sense probe control templates were PCR amplified with a forward primer containing a T7 promoter site (taatacgactcactatagggatgcattatcctaac for *ERF109*; taatacgactcactatagggatggtgaagcaagcg for *ERF018*). Reverse primers were as above. *CLE41* positive control and methods for probe labelling and *in situ* hybridization were as used in [7], and based on the method described in [65].

### Analysis of vascular tissue

Analysis of vasculature tissue in thin sections, was carried out as described previously [66]. For hand cut sections, tissue was stained with either aqueous 0.02% Toluidine Blue or 0.05M Aniline blue in 100 mM Phosphate buffer (pH 7.2). Cell counts were carried out using thin sections of 10 week old stems on ≥10 biological replicates. Cells included were protoxylem (marking the inner part of the bundle), phloem cap cells (marking the outer part of the bundle) and all vascular cell types between. An area of secondary growth is reported to be present up to 2.4 mm above the upper rosette leaf [39]. Consequently, sections were taken 10 mm above the upper rosette leaf to avoid the secondary growth region. Statistical analysis (ANOVA) was carried using SPSS statistical analysis software using an LSD post-hoc test.

### Accession numbers

AGI accession numbers for the genes used in this study are as follows: At3g24770 (*CLE41*), At5g61480 (*PXY*), At4g34410 (*ERF109*), At1g28370 (*ERF11*), At5g61600 (*ERF104*), and At1g74930 (*ERF018*), At4g17500 (*AtERF1*), At5g47220 (*ERF2*), At5g47230 (*ERF5*), At4g17490 (*ERF6*), At4g11280 (*ACS6*), At1g54490 (*EIN5*), At3g15770 (*ETO1*), At5g65800 (*ETO2*), At1g66340 (*ETR1*), At5g03280 (*EIN2*), At1g46480 (*WOX4*).

## Supporting Information

Figure S1 Verification of microarray data using qRT-PCR. qRT-PCR showing expression of *ERF109*, *ERF018* and *ERF1* from tissue equivalent to that used in microarray experiments (the centre of 5 week inflorescence stems), normalised to *ACT2* (A) or *18SrRNA* (B). *expression significantly different from wild type controls (*p*<0.0001). Samples were measured in technical triplicates on biological triplicates.
(TIF)

Figure S2 Reduced *ERF018* expression in Salk_109440 line. qRT-PCR showing level of *ERF018* expression in Salk_109440 inflorescence stems compared to wild type plants. *expression significantly different from wild type controls (*p*<0.05). Samples were measured in technical triplicates on biological triplicates.
(TIF)

Figure S3 Increased vascular cell divisions in *eto2* mutants. (A) and (B) Transverse sections of toluidine blue stained wild type (A), and *eto2* (B) 10 week inflorescence stems. *eto2* mutants have more procambium than wild type (compare middle panels), and initiate secondary growth (arrowheads) between vascular bundles where absent in wild type. Scales are 100 µm (upper panels), 50 µm (middle panels) or 25 µm (lower panels). vb is vascular bundle, if interfascicular region.
(TIF)

Figure S4 Interfascicular tissue in *eto1 erf109 erf1* mutant combinations. (A) Col, (B) *erf109 erf1*, interfascicular tissue from 10 week old inflorescence stems with a clearly defined endodermis. (C) In *eto1* stems vascular cell divisions have been initiated with phloem derived from the divisions (arrowheads). (D) In *eto1 erf109 erf018* this phenotype is suppressed. Scales are 25 µm.
(TIF)

Figure S5 Toluidine blue stained transverse sections of inflorescence stem vascular bundles and hypocotyls at 10 weeks. (A) Combinations of *pxy* and ethylene signalling mutant inflorescence stems. *ein5* and *etr1-3d* are similar to wild type. *pxy ein5* and *pxy etr1-3d* are smaller than *pxy* and *etr1*/*ein5* lines, respectively. In extreme cases (lower panels), *pxy etr1-3d* and *pxy ein5* vascular bundles are extremely small. Scale bars are 50 µm. (B) Transverse sections through hypocotyls show that compared to wild type, *pxy* mutants have disrupted organisation due to loss of orientation of cell division. *ein5* and *etr1-3d* are smaller than wild type but retain ordered vascular tissue. *pxy ein5* and *pxy etr1-3d* hypocotyls are severely reduced in size and lack organisation like *pxy* mutants. Scale bars are 100 µm.
(TIF)

Figure S6 Quantitative analysis of vascular cell division in *pxy*, *ein5* and *etr1* mutant combinations. (A) to (B) Number of cells per vascular bundle at the base of the inflorescence stem of 10 week plants. *pxy etr1-3d* plants compared to *pxy*, *etr1-3d* and wild type controls (A). *pxy ein5* plants compared to *pxy*, *ein5* and wild type controls (B). (C) Hypocotyl diameter (mm) of *pxy etr1-3d* plants at 10 weeks compared to controls. (D) Hypocotyl diameter (mm) of *pxy ein5* plants at 10 weeks compared to controls. α is significantly smaller than Col (*p*<0.01), β is significantly smaller than *pxy* (*p*<0.05), Error bars are standard error.
(TIF)

Figure S7 Expression of PXY signalling components in mutant backgrounds determined by qRT-PCR. qRT-PCR showing expression of *PXY*, *CLE41*, *CLE42* and *WOX4* in inflorescence stem tissue, normalised to *18SrRNA*. Expression did not differ significantly from wild type controls in *ein2* mutants (upper panel), or *erf109 erf018* lines (lower panel). In inflorescence stems subjected to a 16 hour ethylene treatment, *CLE41*, *CLE42* and *WOX4* expression was unchanged, but *PXY* expression was increased (*p<0.05). Samples were measured in technical triplicates on biological triplicates.
(TIF)

Table S1 AP2/ERF family members and ethylene biosynthetic enzyme with differential expression levels in *pxy* versus Col microarray data.
(DOC)

Table S2 qRT-PCR oligos.
(DOC)
