# Supplementary figures and images for: Plant Vascular Cell Division Is Maintained by an Interaction between PXY and Ethylene Signalling
Source: PLoS Genet. 2012 Nov 15;8(11):e1002997. doi: 10.1371/journal.pgen.1002997 (PMC3499249; doi:10.1371/journal.pgen.1002997)

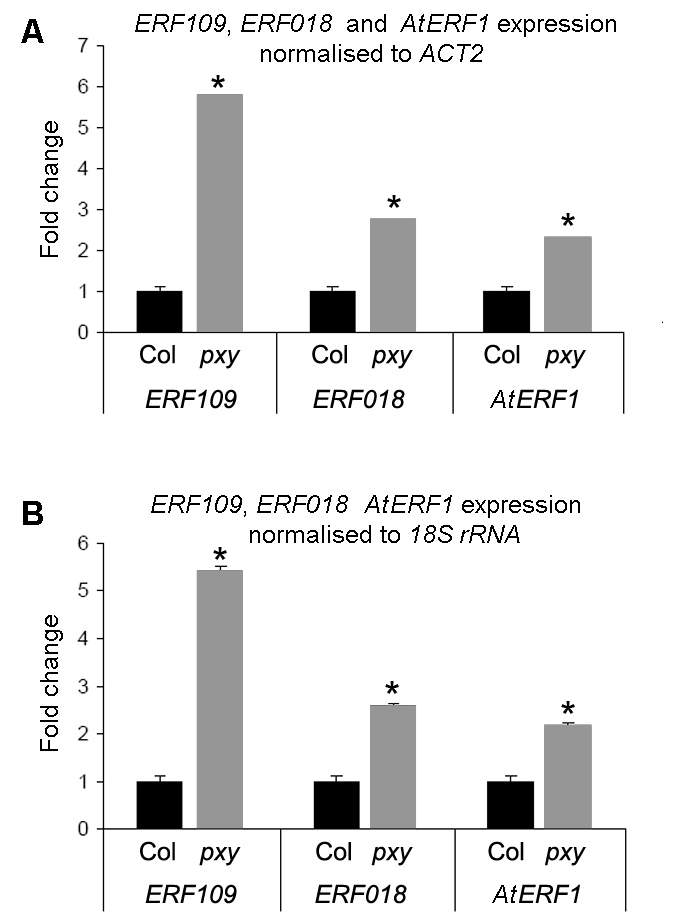

Supplement: Figure S1 — Verification of microarray data using qRT-PCR. qRT-PCR showing expression of ERF109, ERF018 and ERF1 from tissue equivalent to that used in microarray experiments (the centre of 5 week inflorescence stems), normalised to ACT2 (A) or 18SrRNA (B). *expression significantly different from wild type controls (p<0.0001). Samples were measured in technical triplicates on biological triplicates. (TIF) [file pgen.1002997.s001.tif]

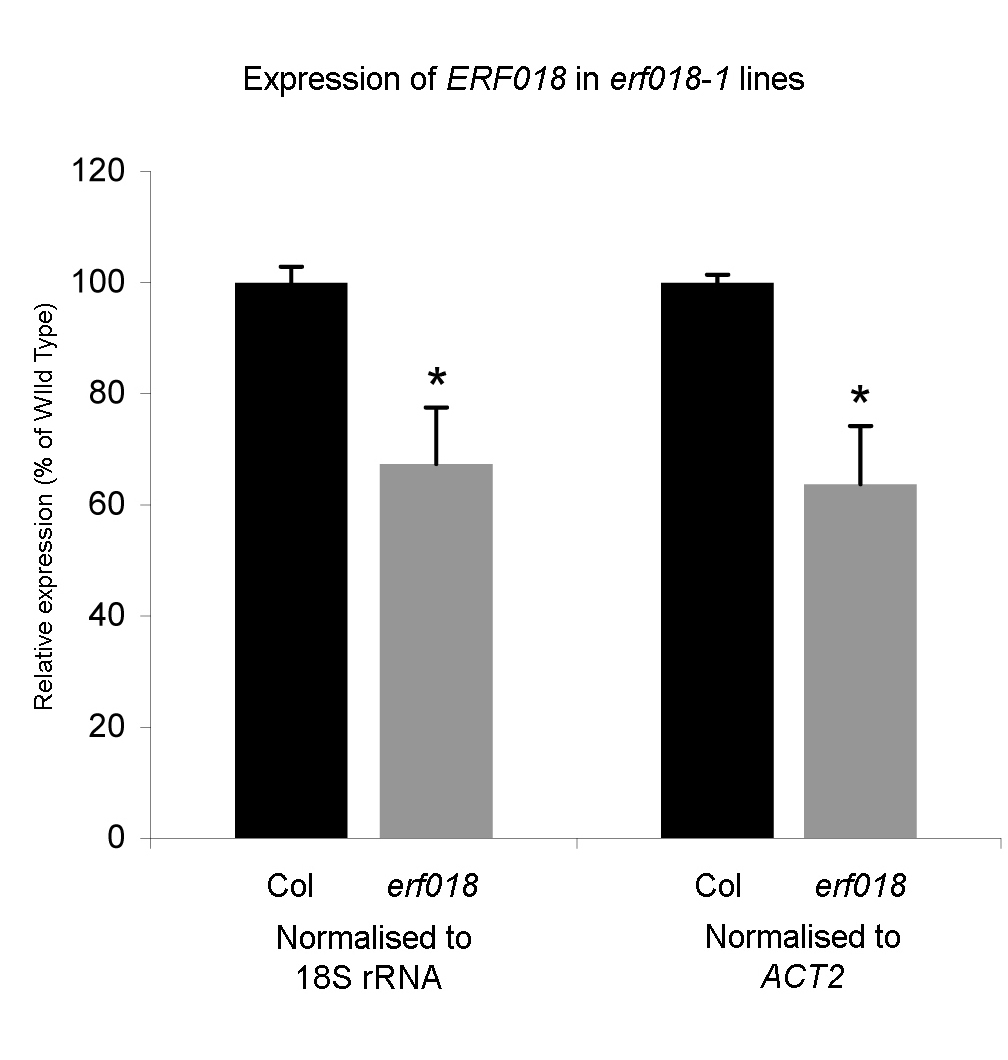

Supplement: Figure S2 — Reduced ERF018 expression in Salk_109440 line. qRT-PCR showing level of ERF018 expression in Salk_109440 inflorescence stems compared to wild type plants. *expression significantly different from wild type controls (p<0.05). Samples were measured in technical triplicates on biological triplicates. (TIF) [file pgen.1002997.s002.tif]

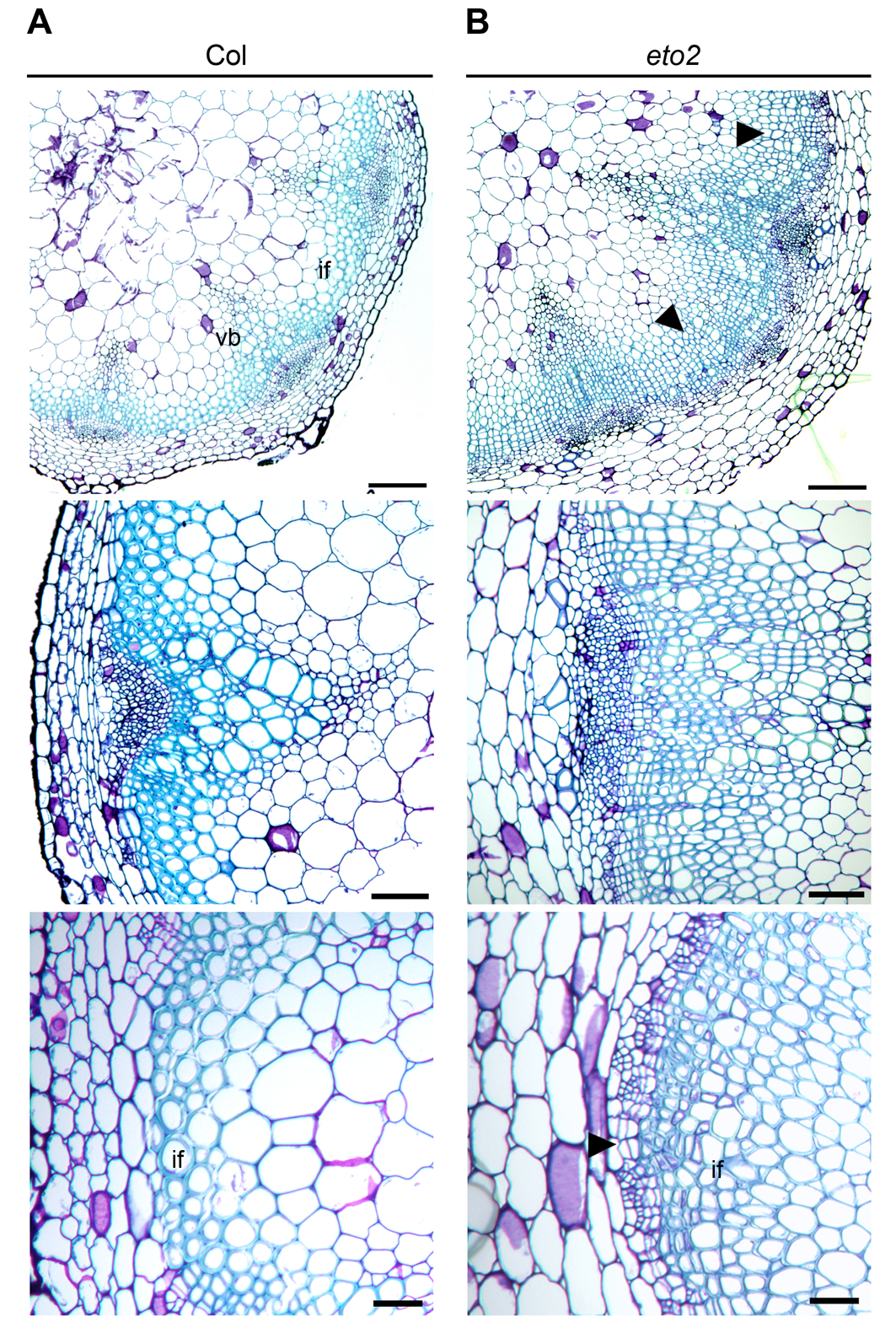

Supplement: Figure S3 — Increased vascular cell divisions in eto2 mutants. (A) and (B) Transverse sections of toluidine blue stained wild type (A), and eto2 (B) 10 week inflorescence stems. eto2 mutants have more procambium than wild type (compare middle panels), and initiate secondary growth (arrowheads) between vascular bundles where absent in wild type. Scales are 100 µm (upper panels), 50 µm (middle panels) or 25 µm (lower panels). vb is vascular bundle, if interfascicular region. (TIF) [file pgen.1002997.s003.tif]

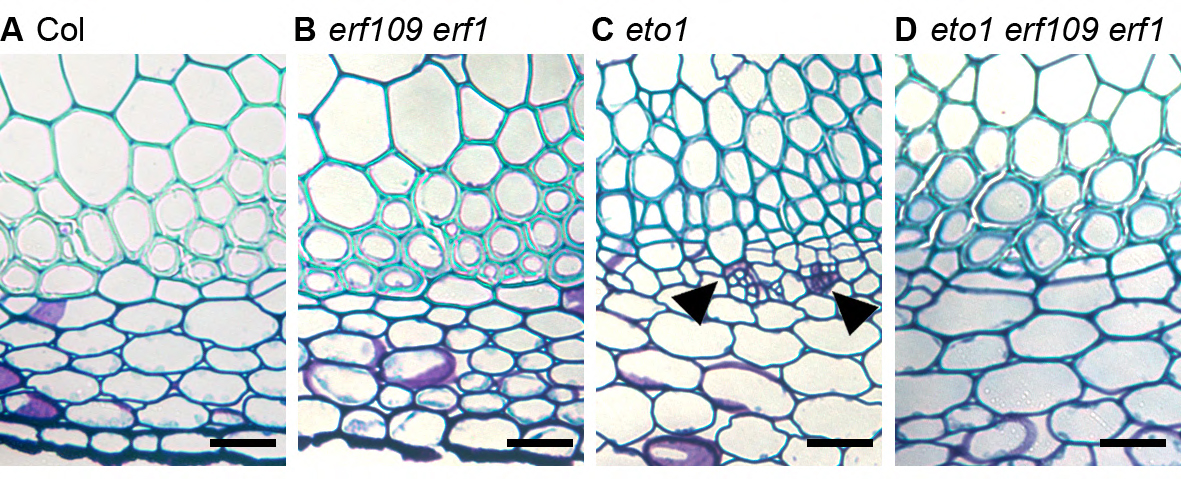

Supplement: Figure S4 — Interfascicular tissue in eto1 erf109 erf1 mutant combinations. (A) Col, (B) erf109 erf1, interfascicular tissue from 10 week old inflorescence stems with a clearly defined endodermis. (C) In eto1 stems vascular cell divisions have been initiated with phloem derived from the divisions (arrowheads). (D) In eto1 erf109 erf018 this phenotype is suppressed. Scales are 25 µm. (TIF) [file pgen.1002997.s004.tif]

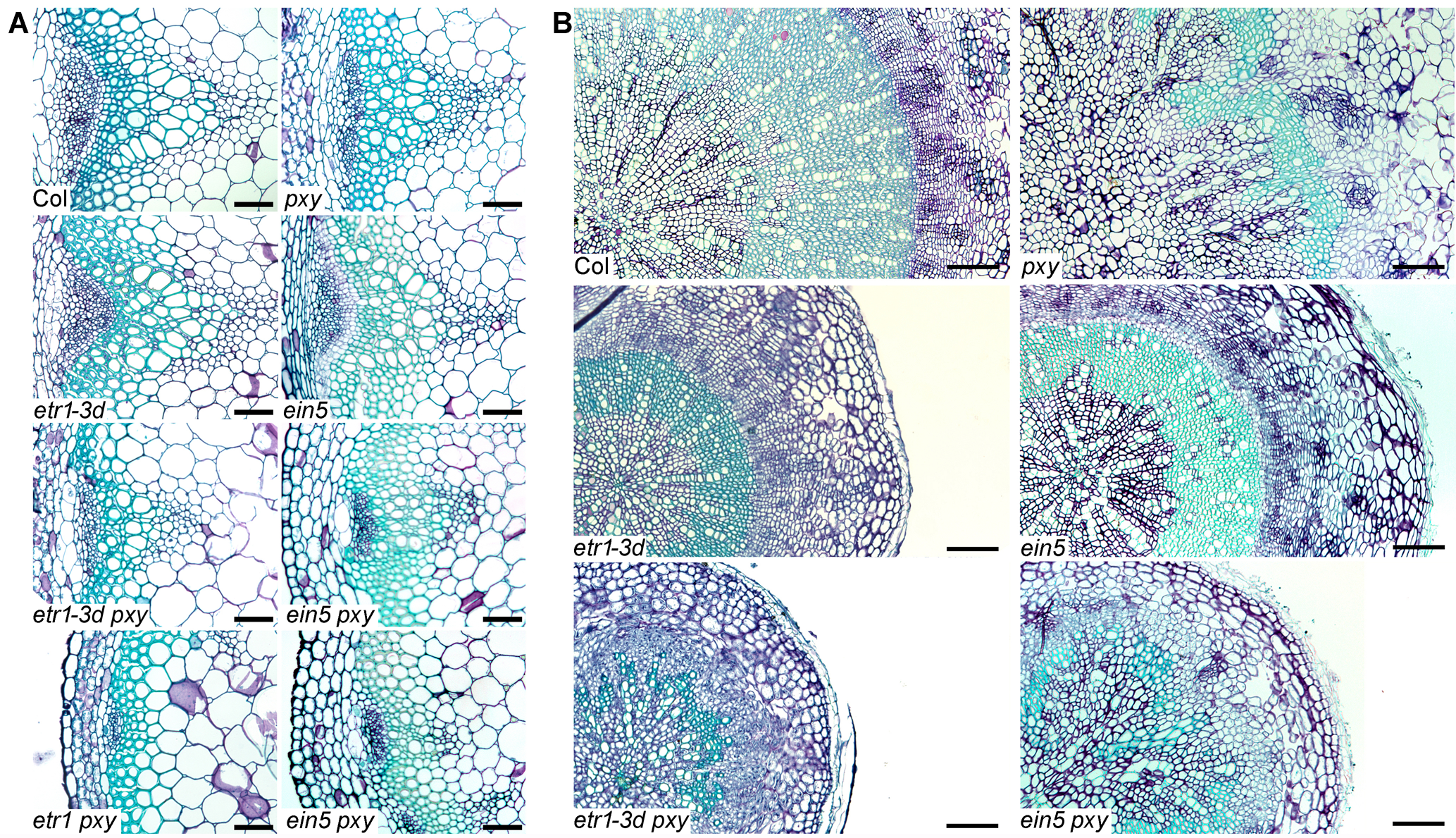

Supplement: Figure S5 — Toluidine blue stained transverse sections of inflorescence stem vascular bundles and hypocotyls at 10 weeks. (A) Combinations of pxy and ethylene signalling mutant inflorescence stems. ein5 and etr1-3d are similar to wild type. pxy ein5 and pxy etr1-3d are smaller than pxy and etr1/ein5 lines, respectively. In extreme cases (lower panels), pxy etr1-3d and pxy ein5 vascular bundles are extremely small. Scale bars are 50 µm. (B) Transverse sections through hypocotyls show that compared to wild type, pxy mutants have disrupted organisation due to loss of orientation of cell division. ein5 and etr1-3d are smaller than wild type but retain ordered vascular tissue. pxy ein5 and pxy etr1-3d hypocotyls are severely reduced in size and lack organisation like pxy mutants. Scale bars are 100 µm. (TIF) [file pgen.1002997.s005.tif]

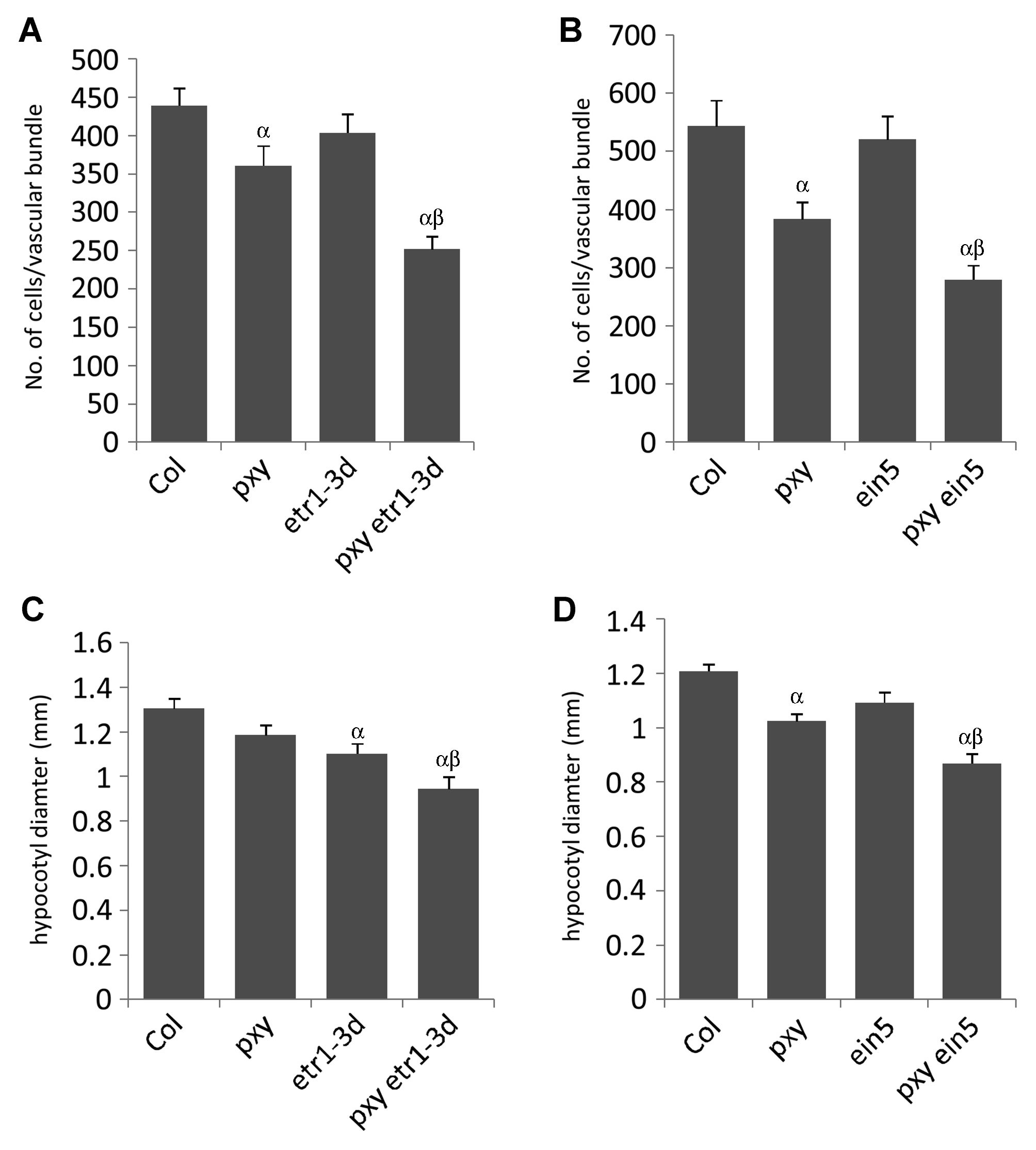

Supplement: Figure S6 — Quantitative analysis of vascular cell division in pxy, ein5 and etr1 mutant combinations. (A) to (B) Number of cells per vascular bundle at the base of the inflorescence stem of 10 week plants. pxy etr1-3d plants compared to pxy, etr1-3d and wild type controls (A). pxy ein5 plants compared to pxy, ein5 and wild type controls (B). (C) Hypocotyl diameter (mm) of pxy etr1-3d plants at 10 weeks compared to controls. (D) Hypocotyl diameter (mm) of pxy ein5 plants at 10 weeks compared to controls. α is significantly smaller than Col (p<0.01), β is significantly smaller than pxy (p<0.05), Error bars are standard error. (TIF) [file pgen.1002997.s006.tif]

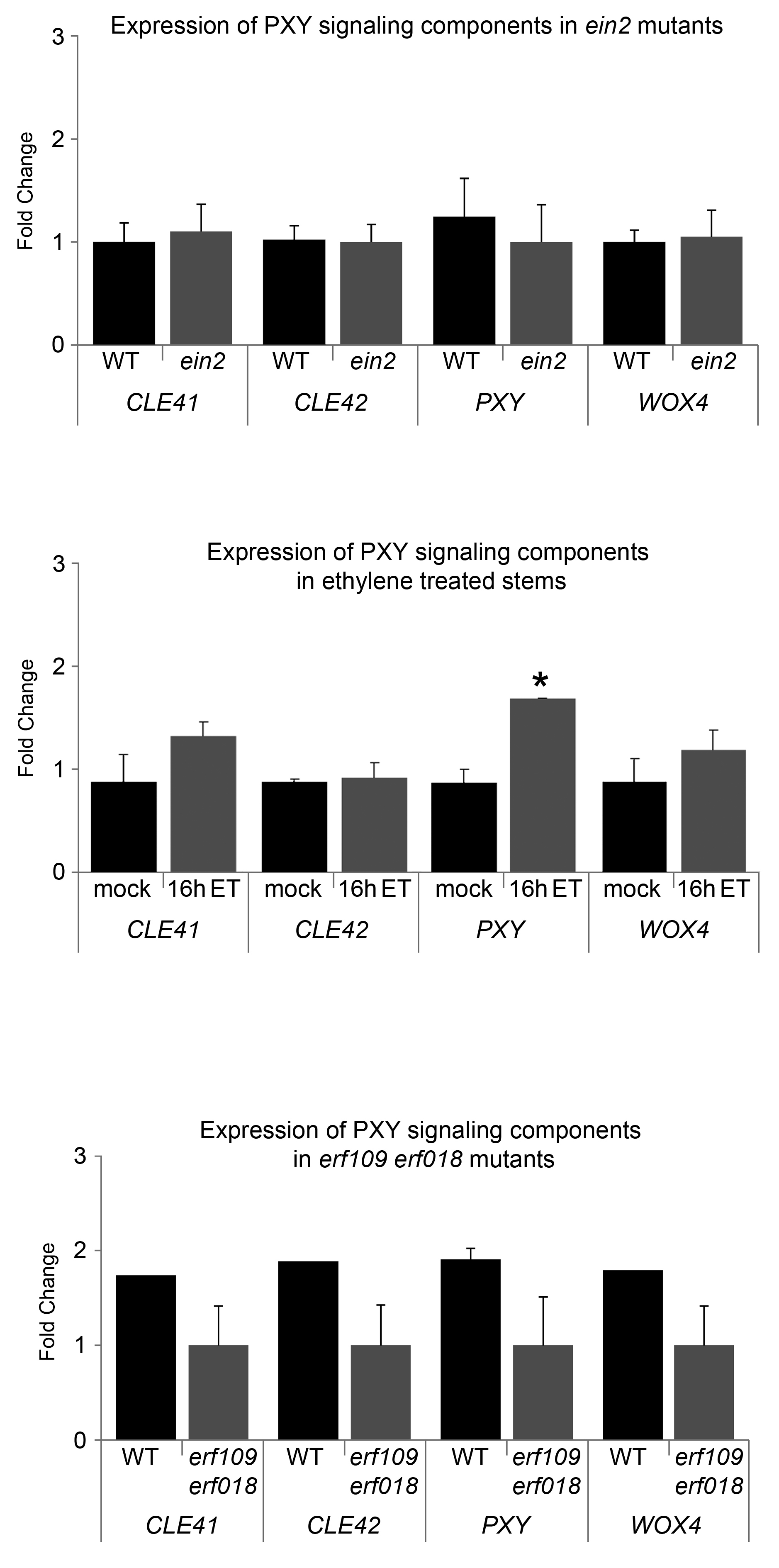

Supplement: Figure S7 — Expression of PXY signalling components in mutant backgrounds determined by qRT-PCR. qRT-PCR showing expression of PXY, CLE41, CLE42 and WOX4 in inflorescence stem tissue, normalised to 18SrRNA. Expression did not differ significantly from wild type controls in ein2 mutants (upper panel), or erf109 erf018 lines (lower panel). In inflorescence stems subjected to a 16 hour ethylene treatment, CLE41, CLE42 and WOX4 expression was unchanged, but PXY expression was increased (*p<0.05). Samples were measured in technical triplicates on biological triplicates. (TIF) [file pgen.1002997.s007.tif]
