# Supplementary material for: Plant Vascular Cell Division Is Maintained by an Interaction between PXY and Ethylene Signalling
Source: PLoS Genet. 2012 Nov 15;8(11):e1002997. doi: 10.1371/journal.pgen.1002997 (PMC3499249; doi:10.1371/journal.pgen.1002997)
Supplement: Table S1 — AP2/ERF family members and ethylene biosynthetic enzyme with differential expression levels in pxy versus Col microarray data. (DOC) [file pgen.1002997.s008.doc]

Supplementary Table S1. AP2/ERF family members and ethylene biosynthetic enzyme with differential expression levels in *pxy* vs Col microarray data.

| **AGI No.** | **Generic ERF No.** | **Other Names** | **Fold Change (*pxy/*WT)** | **p value** |
| --- | --- | --- | --- | --- |
| At4g34410 | AtERF#109 | Redox Responsive Transcription Factor 1 (RRTF1) | 4.27 | 0.0002 |
| At1g28370 | AtERF#076 | ERF11 | 3.01 | 0.0085 |
| At5g61600 | AtERF#104 |  | 2.83 | 0.0004 |
| At1g74930 | AtERF#018 | ORA47 | 2.80 | 0.0013 |
| At5g05410 | AtERF#045 | DREB2A | 2.09 | 0.0007 |
| At4g17500 | AtERF#100 | AtERF1 | 1.99 | 0.0046 |
| At5g47220 | AtERF#101 | ERF2 | 1.90 | 0.0055 |
| At5g44350 | N/A | ethylene-regulated nuclear protein (ERT2-like) | 1.83 | 0.0048 |
| At5g47230 | AtERF#102 | ERF5 | 1.65 | 0.0076 |
| At4g17490 | AtERF#103 | ERF6 | 1.65 | 0.0198 |
| At5g25190 | AtERF#003 |  | 1.58 | 0.0037 |
| At1g19210 | AtERF#017 |  | 1.57 | 0.0804 |
| At4g28140 | AtERF#054 | QRAP2 | 1.55 | 0.0093 |
| At4g11280 | N/A | ACC synthase (AtACS6) | 2.55 | 0.0010 |
