# Supplementary material for: Plant Vascular Cell Division Is Maintained by an Interaction between PXY and Ethylene Signalling
Source: PLoS Genet. 2012 Nov 15;8(11):e1002997. doi: 10.1371/journal.pgen.1002997 (PMC3499249; doi:10.1371/journal.pgen.1002997)
Supplement: Table S2 — qRT-PCR oligos. (DOC) [file pgen.1002997.s009.doc]

**Supplemental Table S2.** qRT-PCR oligos.

| **Oligo Name** | **Sequence (5'-3')** | **Used for** |
| --- | --- | --- |
|  |  |  |
| AtERF#109-F | CAGTTGAAGCAGAGCAATGG | qRT-PCR |
| AtERF#109-R | CCATTCCCAAAATCCATCAT | qRT-PCR |
| AtERF#018-F | TTGATTCCTTCTCCGACGAC | qRT-PCR |
| AtERF#018-R | TCCTCCGTAATCTTCGATGG | qRT-PCR |
| AtERF1-F | CGGTTCGAATCAAGTCCAAG | qRT-PCR |
| AtERF1-R | CACCGTTCTCCTCTTCTTCG | qRT-PCR |
| AtERF11-F | TAAGGCTGGGATGATGGTGT | qRT-PCR |
| AtERF11-R | AAAACCACACGTCGTCCTTC | qRT-PCR |
| ACS6-RT-F | ACGAGACGGTTGCTTTCTGT | qRT-PCR |
| ACS6-RT-R | GGCTTCCACCGTAATCTTGA | qRT-PCR |
| PXY1_RTF | AACCTAGCAATATCCTCCTCGAC | qRT-PCR |
| PXY1_RTR | GGTTCCACCGATCTTTTTCC | qRT-PCR |
| qRT-wox4f | TCACGACCACTGGTGTCTTT | qRT-PCR |
| qRT-wox4r | CCCAGCTCCTACATGTCCTC | qRT-PCR |
| qCLE41f | TCAAGAGGGTTCTCCTCGAA | qRT-PCR |
| qCLE41r | TGTGCTAGCCTTTGGACGTA | qRT-PCR |
| qCLE42f | ACTTCGCCTGAAGGGAAAAG | qRT-PCR |
| qCLE42r | ATTGGCACCGATCATCTTTC | qRT-PCR |
| 18s rRNA F | CATCAGCTCGCGTTGACTAC | qRT-PCR control |
| 18s rRNA R | GATCCTTCCGCAGGTTCAC | qRT-PCR control |
| qACT2f | GCCATCCAAGCTGTTCTCTC | qRT-PCR control |
| qACT2r | ACCCTCGTAGATTGGCACAG | qRT-PCR control |
|  |  |  |
